# Extreme genetic signatures of local adaptation in a notorious rice pest, *Chilo suppressalis*

**DOI:** 10.1093/nsr/nwae221

**Published:** 2024-08-01

**Authors:** Yan Peng, Kaikai Mao, Hongran Li, Junfen Ping, Jingyun Zhu, Xinye Liu, Zhuting Zhang, Minghui Jin, Chao Wu, Nan Wang, Alexander Yesaya, Kenneth Wilson, Yutao Xiao

**Affiliations:** Shenzhen Branch, Guangdong Laboratory of Lingnan Modern Agriculture, Key Laboratory of Gene Editing Technologies (Hainan), Ministry of Agriculture and Rural Affairs, Agricultural Genomics Institute at Shenzhen, Chinese Academy of Agricultural Sciences, Shenzhen 518120, China; Shenzhen Branch, Guangdong Laboratory of Lingnan Modern Agriculture, Key Laboratory of Gene Editing Technologies (Hainan), Ministry of Agriculture and Rural Affairs, Agricultural Genomics Institute at Shenzhen, Chinese Academy of Agricultural Sciences, Shenzhen 518120, China; Guangxi Key Laboratory of Agro-Environment and Agric-Products Safety, College of Agriculture, Guangxi University, Nanning 530004, China; Shenzhen Branch, Guangdong Laboratory of Lingnan Modern Agriculture, Key Laboratory of Gene Editing Technologies (Hainan), Ministry of Agriculture and Rural Affairs, Agricultural Genomics Institute at Shenzhen, Chinese Academy of Agricultural Sciences, Shenzhen 518120, China; Shenzhen Branch, Guangdong Laboratory of Lingnan Modern Agriculture, Key Laboratory of Gene Editing Technologies (Hainan), Ministry of Agriculture and Rural Affairs, Agricultural Genomics Institute at Shenzhen, Chinese Academy of Agricultural Sciences, Shenzhen 518120, China; School of Life Sciences, Henan University, Kaifeng 475004, China; Shenzhen Research Institute of Henan University, Shenzhen 518000, China; Shenzhen Branch, Guangdong Laboratory of Lingnan Modern Agriculture, Key Laboratory of Gene Editing Technologies (Hainan), Ministry of Agriculture and Rural Affairs, Agricultural Genomics Institute at Shenzhen, Chinese Academy of Agricultural Sciences, Shenzhen 518120, China; Shenzhen Branch, Guangdong Laboratory of Lingnan Modern Agriculture, Key Laboratory of Gene Editing Technologies (Hainan), Ministry of Agriculture and Rural Affairs, Agricultural Genomics Institute at Shenzhen, Chinese Academy of Agricultural Sciences, Shenzhen 518120, China; Shenzhen Branch, Guangdong Laboratory of Lingnan Modern Agriculture, Key Laboratory of Gene Editing Technologies (Hainan), Ministry of Agriculture and Rural Affairs, Agricultural Genomics Institute at Shenzhen, Chinese Academy of Agricultural Sciences, Shenzhen 518120, China; Shenzhen Branch, Guangdong Laboratory of Lingnan Modern Agriculture, Key Laboratory of Gene Editing Technologies (Hainan), Ministry of Agriculture and Rural Affairs, Agricultural Genomics Institute at Shenzhen, Chinese Academy of Agricultural Sciences, Shenzhen 518120, China; Shenzhen Branch, Guangdong Laboratory of Lingnan Modern Agriculture, Key Laboratory of Gene Editing Technologies (Hainan), Ministry of Agriculture and Rural Affairs, Agricultural Genomics Institute at Shenzhen, Chinese Academy of Agricultural Sciences, Shenzhen 518120, China; Shenzhen Branch, Guangdong Laboratory of Lingnan Modern Agriculture, Key Laboratory of Gene Editing Technologies (Hainan), Ministry of Agriculture and Rural Affairs, Agricultural Genomics Institute at Shenzhen, Chinese Academy of Agricultural Sciences, Shenzhen 518120, China; Shenzhen Branch, Guangdong Laboratory of Lingnan Modern Agriculture, Key Laboratory of Gene Editing Technologies (Hainan), Ministry of Agriculture and Rural Affairs, Agricultural Genomics Institute at Shenzhen, Chinese Academy of Agricultural Sciences, Shenzhen 518120, China; Shenzhen Branch, Guangdong Laboratory of Lingnan Modern Agriculture, Key Laboratory of Gene Editing Technologies (Hainan), Ministry of Agriculture and Rural Affairs, Agricultural Genomics Institute at Shenzhen, Chinese Academy of Agricultural Sciences, Shenzhen 518120, China; Lancaster Environment Centre, Lancaster University, Lancaster LA1 4YW, UK; Shenzhen Branch, Guangdong Laboratory of Lingnan Modern Agriculture, Key Laboratory of Gene Editing Technologies (Hainan), Ministry of Agriculture and Rural Affairs, Agricultural Genomics Institute at Shenzhen, Chinese Academy of Agricultural Sciences, Shenzhen 518120, China

**Keywords:** local adaptation, *Chilo suppressalis*, gene flow, cold tolerance

## Abstract

Climatic variation stands as a significant driving force behind genetic differentiation and the evolution of adaptive traits. *Chilo (C.) suppressalis*, commonly known as the rice stem borer, is a highly destructive pest that crucially harms rice production. The lack of natural population genomics data has hindered a more thorough understanding of its climate adaptation, particularly the genetic basis underlying adaptive traits. To overcome this obstacle, our study employed completely resequenced genomes of 384 individuals to explore the population structure, demographic history, and gene flow of *C. suppressalis* in China. This study observed that its gene flow occurred asymmetrically, moving from central populations to peripheral populations. Using genome-wide selection scans and genotype-environment association studies, we identified potential loci that may be associated with climatic adaptation. The most robust signal was found to be associated with cold tolerance, linked to a homeobox gene, *goosecoid* (*GSC*), whose expression level was significantly different in low and high latitudes. Moreover, downregulating the expression of this gene by RNAi enhances its cold tolerance phenotypes. Our findings have uncovered and delved into the genetic foundation of the ability of *C. suppressalis* to adapt to its environment. This is essential in ensuring the continued effectiveness and sustainability of novel control techniques.

## INTRODUCTION

Temperature is one of the fundamental factors that influence species distribution, as it directly impacts physiological functions that drive population growth [1]. In tropical regions, there is limited seasonal variation and consistently high ambient temperatures, unlike higher latitudes where there is noticeable seasonality, daily temperature fluctuations, and cooler temperatures. As such, thermal tolerance of ectotherms can be influenced by these climatic characteristics [2]. Pests can regulate mismatches between the environment and their traits by undergoing fitness shifts, developing tolerance and adaptation [3–6]. Climatic gradients play a significant role in shaping the fitness-related characteristics of pests. These characteristics include their ability to endure low temperatures [7]. Two key factors that contribute to their adaptation to changing climates are diapause and cold tolerance [8–10]. In addition, due to their short generation times and large effective population sizes, pests likely adapt to local climatic conditions [11]. Exploring how pests can adapt to cold environments serves as a fundamental principle for comprehending ecological adaptability [12,13]. One of the major challenges lies in the fact that the genetic foundation that footprint adaptation to climate change remains largely unknown.

At the optimal temperature, the ability of pests to survive, reproduce, and develop is enhanced [14]. However, deviations of temperature values that are either higher or lower than the optimal temperature have more pronounced negative impacts on populations [15]. Investigating genetic adaptation to cold environments in pest species faces challenges, primarily due to difficulties in culturing them under laboratory conditions. These challenges are especially pronounced for pests with long generation times or complex life cycles in high-latitude populations. However, an alternative strategy is to analyze natural populations to identify the genetic basis of adaptation linked to climate characteristics. Genome scans have the potential to offer effective and efficient methods for identifying genetic variations that are linked to adaptation [13,16,17]. Furthermore, knowledge of demographic history and gene flow with geographic barriers can greatly reduce a significant confounding effect in genome scans [12]. When gene flow is extensive, both neutral and adaptive variations are homogenized, and locally adapted alleles may be overwhelmed [18]. In contrast, when gene flow is absent, adapted alleles in local populations evolve independently, and their survival within the population is determined by the interplay of selection and genetic drift [19]. This type of information can be essential for understanding the principles behind limiting adaptation, as well as management practices for range edge populations [20].

*Chilo (C.) suppressalis* Walker (Lepidoptera: Crambidae), commonly known as the rice stem borer, is a prevalent rice pest that poses a significant risk to food security in various parts of Asia [21–23]. Geographically, *C. suppressalis* is commonly distributed across Asia, Oceania, the Middle East, and Europe [24]. In China, it is distributed across Heilongjiang Province to Hainan Island in the tropics located at 50°N and 18°N with mean January temperatures of below −20°C and above 15°C, respectively. This broad distribution suggests that *C. suppressalis* populations possess diverse ecological phenotypes that enable them to adapt to a variety of complex environmental conditions. For instance, *C. suppressalis* mature larvae can enter facultative diapause during the beginning of autumn [25]. These two geographical populations, namely the North China region represented by Beijing and the South China region represented by Fuzhou, displayed notable distinctions in terms of their critical photoperiods and resistance to cold conditions. Populations in the higher-latitude area showed longer critical photoperiods and higher levels of cold hardiness [26]. The ability of *C. suppressalis* to tolerate cold temperatures is not affected by the insect's diapause state [27,28], although the body weights of overwintering larvae were significantly affected by the temperature treatments [29,30]. These results indicated that body weights may vary according to geographical location and conditions. *C. suppressalis*, which possesses varying traits across distinct geographical populations, is an excellent model organism for investigating climate adaptation. However, limited availability of genomic resources for *C. suppressalis* has seriously impeded progress in understanding these important genetic mechanisms.

Our goal is to enhance the knowledge and understanding of pest genome evolution at varying latitudes by delving into the population genetics of *C. suppressalis*. To accomplish this, we initially resequenced 384 *C. suppressalis* individuals collected from China's primary geographic regions extending across various latitudes. Using this information, we evaluated the population genetic structure, demographic history, and gene flow of *C. suppressalis*. We also conducted a genome-wide scan to pinpoint genes and genomic regions that may have adapted to the climatic conditions. Since selection could have occurred at different stages during the populations’ colonization history, we implemented multiple methods to identify both recent and ancient signals of selection. By studying genotype-environment associations with environmental factors, we additionally discovered candidate loci that could be linked to climatic adaptation, with the most robust signal being associated with cold temperatures. We noted that reducing the expression of this gene enhances its cold tolerance. This discovery fills a fundamental gap in the pest adaptive evolution story and furthers our appreciation of the mechanism underpinning rapid adaptation.

## RESULTS

### Genomic variation and population structure

**Figure 1.**
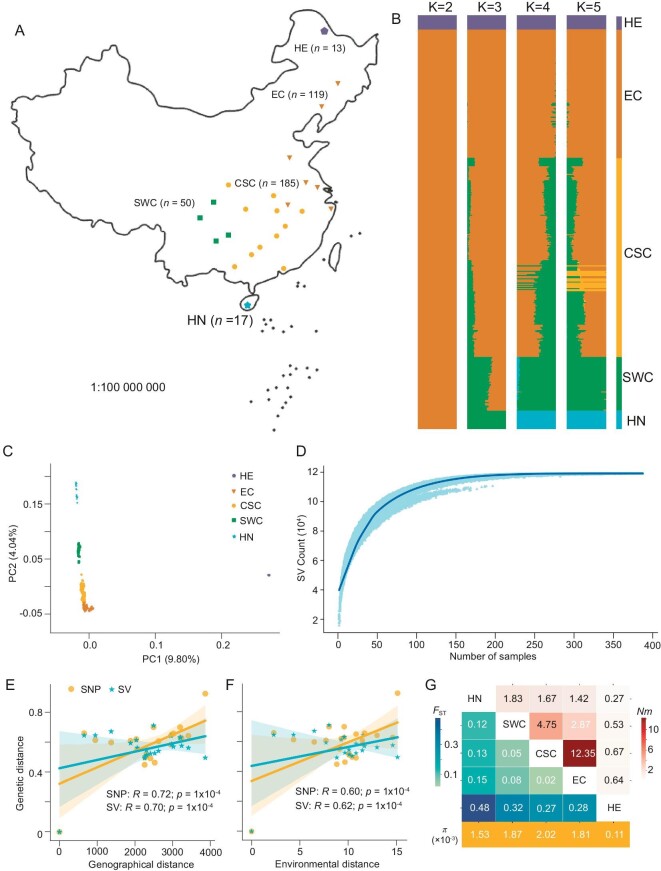
Collection locations and population-level genomic analyses of *Chilo suppressalis* in China. (A) The spatial patterns of 24 sampling locations. (B) Ancestry analysis for K = 2–5 using SNPs. Five populations are labeled: HeiHe (HE), East Central (EC), Central South China (CSC), Southwest China (SWC), and Hainan (HN). (C) PCA plot of the first two components (PC1 and PC2) using SNPs. (D) Simulations were conducted to observe how the number of SVs detected increases with the addition of accessions by repeating the process 1000 times. The light green data points were fitted with a blue curve to represent the trend. (E and F) Geographic and environmental distances explain genetic variation in *C. suppressalis* using SNPs and SVs. (G) Pairwise comparisons of fixation index (*F*_ST_, lower triangle), corresponding gene flow *Nm* (upper triangle) and diversity (π) values between different populations. Review drawing number: GS 京 (2024)1256.

To understand the genetic basis of local adaptation in *Chilo suppressalis*, we obtained 384 samples from 24 different locations across China (Fig. 1A), representing a wide range of geographic distributions. Basically, the clean reads were mapped to the *C. suppressalis* genome [21], with an average mapping rate of approximately 93.43%, an average coverage of 85.05%, and a depth of 26.22× (Table S1). Based on these data, we detected a total of 5 280 742 high-quality single nucleotide polymorphisms (SNPs).

To obtain a better understanding of genetic relationships among populations within China, two complementary approaches were employed to investigate the population structure of *C. suppressalis*: (i) ancestry analysis with fastStructure software and (ii) principal component analysis (PCA). With K = 2, the Heihe (HE) population formed one cluster. From K = 3 onward, the Hainan (HN) population became distinct from the others. With K = 4 and K = 5, the East Central (EC) and Southwest China (SWC) populations were identified as separate genetic clusters, whereas the Central South China (CSC) population exhibited indications of mixed ancestry (Fig. 1B; Figs S1–S2). We obtained similar findings through PCA (Fig. 1C). The first principal component (PC), explaining 9.80% of the variance, distinguished the HE population. The second PC with 4.04% of variance separated the HN population from the remaining populations.

Structural variations (SVs) have been linked to genome evolution [31]. Focusing on the SVs that were detected by short reads analyses, a final set of 119 022 SVs, ranging from 50 bp to 1 Mb, included 12 876 duplications (DUP), 8904 inversions (INV), 32 insertions (INS), 72 277 deletions (DEL), and 24 933 translocations (TRA) (Fig. S3). By randomly sampling accessions iteratively and modeling the SV size, we determined that the SV count was relatively limited in the *C. suppressalis* population (Fig. 1D). This suggested that our SV detection was comprehensive and nearly complete. The population structure based on these identified SVs exhibited similar results to those derived from SNPs (Fig. S4).

To investigate the genetic differentiation patterns using isolation-by-distance (IBD) and isolation-by-environment (IBE) analyses with SNPs and SVs, we computed the Euclidean distance derived from environmental factors, downloaded from public environmental databases, as a measure of environmental distance. The populations were selected from 24 geographic regions (>5 individuals per population). The genetic distance between locations, measured by *F*_ST_/(1–*F*_ST_) [32], strongly correlated with both geographic and environmental distances when analyzing SNPs and SVs (Fig. 1E–F). The results indicated that the major factor shaping genome-wide variation was IBD. This result was not unexpected, as environmental adaptation was anticipated to affect only a small fraction of the genome [33]. Considering the unequal population sizes, we randomly selected 10 individuals from each population to estimate *F*_ST_ and assess genetic diversity. Consequently, to quantify the identified genetic differentiation, we performed pairwise *F*_ST_ analysis according to the results of the population structure (Fig. 1G). A pairwise *F*_ST_ heatmap and *Nm* results revealed a strong correlation between the HN, SWC, CSC, and EC populations, suggesting that these populations may experience gene flow (*Nm* >1) or recent divergence. In contrast, the HE population exhibited significant genetic divergence from the other populations (Fig. 1G). The genetic diversity of the peripheral populations (HE and HN) was relatively lower compared to the central populations (EC, SWC, and CSC) (Fig. 1G; Figs S6–S7). Overall, our results indicated relatively weak genetic differentiation between the HN, SWC, CSC, and EC populations, except the HE population. The results suggested that these geographical populations, excluding the HE population, may have undergone recent divergence.

### Demographic history and gene flow in the *C. suppressalis* populations

**Figure 2.**
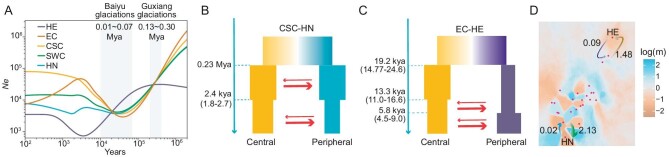
Demographic history and gene flow of *Chilo suppressalis* populations. (A) Changes in effective population size (*Ne*) over time inferred by five geographical populations using SMC++. The gray shading shows the Baiyu Glaciations (0.01–0.07 Mya) and Guxiang Glaciations (0.13–0.30 Mya). (B and C) Demographic history simulated by fastsimcoal2. The arrow indicates the direction of gene flow. Point estimates of parameters and their corresponding 95% confidence intervals are reported in Table S3. (D) The colors display the EEMS representing migration barriers (orange) and channels (cyan). The purple dots represent the sample points. The direction of gene flow is demonstrated by the arrows using fastsimcoal2. The thickness of the line represents the population migration rates (4*N_e_m*).

To evaluate the demographic history of *C. suppressalis*, specifically regarding the occurrence of bottleneck events during the colonization process, we first investigated past fluctuations in the effective population size (*N*_e_) of each population (*n* = 10 per population) using SMC++ [34]. The HE population had the lowest historical *N*_e_ and exhibited a notable distinction from the other populations. Furthermore, our analysis revealed that the inferred *N*_e_ was influenced by the Guxiang Glaciation (0.13–0.30 Mya) and Baiyu Glaciation (0.01–0.07 Mya) events in the *C. suppressalis* population (Fig. 2A). The low *F*_ST_ values observed among the CSC, EC, and SWC populations, suggested that gene flow may have occurred. We also investigated gene flow between the two peripheral populations (HE and HN, *n* = 10 per population) and the central populations (EC and CSC, *n* = 10 per population) employing multiple models in two populations (CSC-HN, *n* = 9; EC-HE, *n* = 13) using fastsimcoal2 [35]. The most suitable model indicated that gene flow occurred from the central populations to the peripheral populations, and that the gene flow was asymmetric (Fig. 2B and C; Figs S8–S10 and Tables S2–S3). Thereafter, we employed EEMS to estimate an effective migration surface [36], which allowed us to visualize the gene flow patterns in different populations. The EEMS analysis clearly demonstrates a significant reduction in gene flow between peripheral populations and central populations (Fig. 2D). Although gene flow from central to peripheral populations has a negative impact on fitness, it is important to note that gene flow can also have positive effects on peripheral populations [20,37].

### Selective sweeps related to adaptation in the *C. suppresassalis* genome

**Figure 3.**
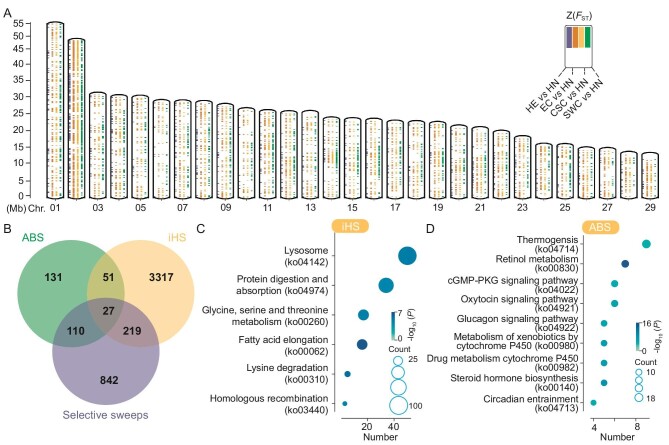
Evidence of selective sweeps in *Chilo suppressalis*. (A) Genome-wide screening of the outlier regions based on population divergence [z(*F*_ST_)]. The different colors represent the outlier regions by comparing other populations with the low-latitude population (HN population). (B) Venn diagram shows the number of genes identified by ABS, iHS, and selective sweeps. (C and D) The scatterplot represents the KEGG categorization of identified genes (iHS and ABS) in significant pathways. The color of the circle displays the −log10(*P* ) value. The size of the circle displays the number of these recent and ancient positive selection signals in the enriched pathway.

The *C. suppresassalis* populations can adapt to various environments through the process of natural selection (Table S4). To determine the genomic loci that may have facilitated climate adaptation, we first computed the z transformation of *F*_ST_ by comparing other populations with the low latitude population (HN population). The outlier regions spanned 13.97 Mb, 34.86 Mb, 35.22 Mb, and 36.59 Mb [HN vs. (HE, EC, CSC, SWC)] (Fig. 3A). However, a neutral process has the potential of leading to the emergence of diverse regions with increased differentiation. To ensure that we minimize the impact of neutral processes, it is crucial to employ additional methods for detecting regions of selective sweep. Genes determined by two or more methods [top 5%, Z(*F*_ST_), u statistics, composite-likelihood ratio (CLR)] are considered as candidate genes. We have identified a total of 1198 genes (Fig. 3B; Fig. S11 and Table S5). We also annotated these genes by mapping to the Kyoto Encyclopedia of Genes and Genomes (KEGG) pathways. Eighteen KEGG pathways were significantly enriched, including GABAergic synapse (ko04727), circadian rhythm (ko04713), and longevity regulating pathway (ko04211) (Fig. S12 and Table S6).

To uncover additional evidence of recent positive selection, we utilized iHS statistics, which measured the extended haplotype homozygosity score of the ancestral and derived alleles at each genetic locus. This method enables the detection of ongoing or recent positive selection, as older selective signals would have been disrupted by recombination over time [38–40]. Older signals could provide insights into the evolutionary history of ancestral populations [41], and we used the ancestral branch statistic (*ABS*) to detect more ancient positive selection signals. A total of 3614 and 319 candidate genes were identified by iHS and ABS, respectively (Fig. 3B; Tables S7 and S9). KEGG enrichment analysis revealed that these recent positive genes were significant in fatty acid elongation (ko00062) (Fig. 3C; Table S8). However, these ancient positive selection signals were significantly enriched in retinol metabolism (ko04714) (Fig. 3D; Table S10). Interestingly, circadian entrainment (ko04713) was enriched by ABS, not iHS, which may indicate that circadian pathways may play an important role in ancestral selection.

### Genomic variants linked to environmental factors

**Figure 4.**
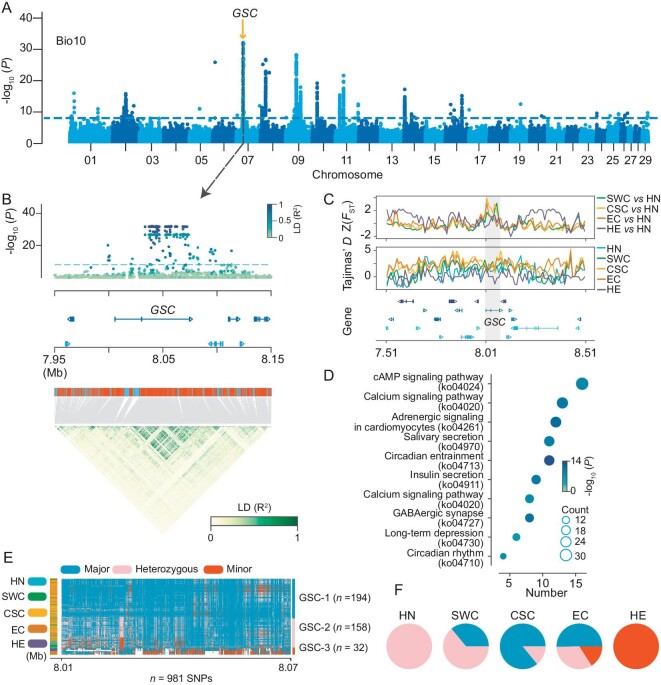
Variations in the genome linked to BIOCLIM variables. (A) Manhattan plots of the temperature variables (bio10) using LMM models with SNPs. (B) Local Manhattan plots for key environmental factors (bio10), gene positions, and LD heatmaps representing the strong peaks of the adaptive candidate gene (*GSC*). (C) Zoomed-in plot for the candidate region on chromosome 7. The Z(*F*_ST_) and Tajima's *D* were calculated using a 10-kb sliding window. (D) The scatterplot represents the KEGG categorization of these genes identified using BayPass. The color of the circle represents the −log10(*P* ) value. The size of the circle represents the number of genotype- and environment-associated genes in the enriched pathway. (E) Heatmap plots represent the haplotypes found for the *GSC* gene. (F) A pie chart is utilized to display the distribution of haplotype proportions for the *GSC* gene across various populations.

The distribution area of *C. suppresassalis* is broad in China (Table S4). To survive in diverse geographical locations and varying habitats, *C. suppresassalis* is likely to have adaptive evolution mechanisms for local environments. To further investigate the loci driven by climatic gradients, we used two genome-scan methods [BayPass and linear mixed model (LMM)]. BayPass and LMM account for the confounding effects of neutral differentiation among populations. In this analysis, we utilized data collected from WorldClim [http://www.worldclim.org], including latitude and two environmental factors: mean temperature of the warmest quarter (bio10) and precipitation of the driest quarter (bio17) (Fig. S13 and Tables S11–S12). Using BayPass and LMM methods, we identified 20 690 and 3502 SNPs, respectively (Fig. S14). In terms of temperature environmental factors, we found 294 genes associated with bio10 using LMM (Fig. 4A). The most significant signal of association was linked to *goosecoid* (*GSC*), a homeobox gene that functions as a transcription factor involved in morphogenesis [42] (−log *p* of peak SNP = 32.05) (Fig. 4B). The *GSC* gene was the most pleiotropic candidate gene associated with latitude and bio17 environmental factors (Fig. S16). Additionally, we chose to exclude these two peripheral populations (HE and HN) and solely focus on the main central populations. Our findings further support the significant role of the *GSC* gene, which align with the results obtained when analyzing all populations (Fig. S16, Table S13). In the local region, we used SNPs and found that the *GSC* gene had elevated genetic differentiation (Fig. 4C). Furthermore, our findings revealed that the Tajima's *D* values were negative in the HE population, suggesting ongoing adaptation of the *GSC* gene (Fig. 4C). To further test the function of the genes identified by LMM (*n* = 514) and BayPass (*n* = 773) (Tables S14–S17), we also performed KEGG enrichment analysis. We found that the most significant pathways were associated with circadian gene (Fig. 4D, circadian entrainment; ko04713). Moreover, the circadian clock gene (*period; per*) was enriched in this pathway (Table S17). The *per* gene is associated with diapause in other pests [5,43]. We further discovered that significant haplotype variations exist within the *GSC* gene (Fig. 4E). The *GSC* gene's haplotype is not confined to a particular population but varies according to latitude, suggesting significant biological roles (Fig. 4F).

### An extreme genetic signature of local adaptation was associated with cold tolerance

**Figure 5.**
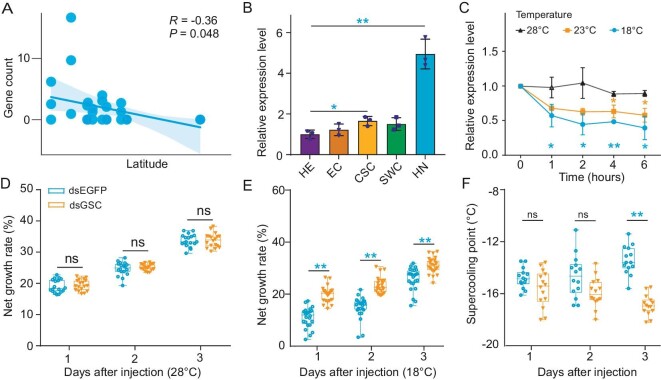
Candidate causative gene and variants for cold tolerance. (A) Scatter plots show the relationship between the standardized read counts and latitude. (B) Quantitative real-time PCR analyses of the *GSC* gene in five *C. suppressalis* populations. The *P* values were calculated using Student's *t* test, *P < 0.05; **P < 0.01. (C) Line chart of points representing the relative expression level of *GSC* genes under different temperatures (18°C, 23°C, and 28°C). The *P* values are calculated using Student's *t* test, *P < 0.05; **P < 0.01. The boxplots show the net growth rate at different temperatures, 28°C (D) and 18°C (E), as well as the supercooling point (F) in *C. suppressalis* after RNAi silencing at 1, 2, and 3 d for dsGSC and dsEGFP. The statistical significance was determined using Student's *t* test, with * indicating a significance level of *P* < 0.05 and ** indicating a significance level of *P* < 0.01.

To determine the biological function of the most significant signal, we conducted a further analysis of 30 transcriptomic datasets from different populations. Our investigation revealed a noteworthy correlation between the expression of the *GSC* gene and latitude (Fig. 5A). This finding was confirmed by quantitative reverse transcription PCR (Fig. 5B). We observed a significant upregulation of *GSC* expression by 1.66- and 4.95-fold in the CSC and HN populations, respectively, compared to the HE population. Furthermore, we investigated the response of *GSC* genes to temperature fluctuations by examining the expression levels of *GSC* genes at various time points under different temperatures (18°C, 23°C, and 28°C). Our findings indicated that the expression of *GSC* was significantly downregulated at 23°C (4 h and 6 h) as well as at 18°C (1 h, 2 h, 4 h, and 6 h) treatment compared to that at 28°C (Fig. 5C).

A further *in vivo* functional study was conducted using RNA interference (RNAi) to silence the *GSC* gene, followed by analyses of larval supercooling points and net growth rates at low temperature to assess the physiological significance of the *GSC* gene associated with resistance to cold conditions in *C. suppressalis* (Fig. S17). dsRNA silencing of the *GSC* gene showed that there was no significant difference at 28°C (Fig. 5D). However, when injected at 18°C, it triggered a significant increase in the net growth rate of larvae after 1, 2 and 3 d of injection (Fig. 5E). Reducing *GSC* expression also resulted in a significant decrease in larval supercooling points at 3 d following dsGSC injection compared with the dsEGFP-injected group (Fig. 5F). These results suggested that *GSC* functions as a downregulating genetic factor for the cold tolerance trait. In general, adaptation may occur by altering the amount of *GSC* gene expression.

## DISCUSSION

The study has provided insights into various aspects of genomic adaptation and evolution through resequenced genomes of *C. suppressalis* populations. Using mitochondria markers (432 bp fragments of the cytochrome b gene), previous studies found that the genetic structure in China separated into five populations [44]. The central China population acted as a transition zone between the northern and southern regions. The *C. suppressalis* populations display significant geographical differences in ecological preference [45]. These results concurred with those of nuclear genome. By studying SNPs and SVs in the nuclear genome, we found that the peripheral populations (HE and HN) have a relatively stronger difference to the central populations (EC, SWC, and CSC). HE is located at the eastern end of the Greater Khingan Mountains and the northern part of the Lesser Khingan Mountains. The area is characterized by continuous mountain ranges and crisscrossing valleys. Therefore, the region can have unique geographical conditions that lead to genetic differentiation. The species exhibits distinct phenotypic traits, such as having only one generation per year as observed in the HE population, while the Harbin population (collection site in Harbin, Heilongjiang Province in China) has two generations annually. The fact is that significant genetic differentiation in peripheral populations has also been observed in other insects (*F*_ST_ >0.3) [16]. For instance, in a different insect species, subspecies from distinct groups exhibited an average pairwise *F*_ST_ (allelic fixation index) of 0.42 [46]. These observations suggest that the HE population may have evolved differently from the central population (Fig. 2B and C). Future research should delve into inferring ancestral variation states to determine whether they originate from standing genetic variation or novel mutations under divergent selection, similar to the example mentioned [16].

Natural populations of *C. suppressalis* are ideal models to investigate the adaptative traits in different climates. *C. suppressalis* has an extensive geographical distribution, and these adaptation processes have left an imprint on the genome. We also found that circadian entrainment (ko04713), an important pathway for insect environmental adaptation [47,48], was enriched by ABS. These results indicated that these genes were selected early in the colonization history. Another pathway, fatty acid elongation (ko00310), was enriched by iHS. However, further investigation is needed to identify if there has been recent selection, particularly in a rapid environmental change, as the progression of selection is based on the sorting of ancestral standing variation and not entirely dependent on *de novo* mutations. However, genome scans relating to local adaptation still face challenges to be accurately accounted for demographic histories [12]. As such, there is a continuous requirement for novel simulation methods to assess their efficacy across varying scenarios such as different genetic architectures, demographic histories, and selection strengths [49]. Furthermore, it is crucial to integrate various approaches into a comprehensive statistical framework.

We further conducted genotype-environment association analysis by focusing on the strongest associated signal in the *GSC* gene using SNPs. Moreover, we did not detect the nonsynonymous SNPs in the *GSC* region. The reason might be that SVs account for an equal or greater amount of phenotypic variation compared to SNPs [31,50]. Thus, further investigation is required to ascertain the genetic mechanisms and causal genetic variants involved, potentially leveraging multi-omics sequencing technology. Bearing in mind that cold tolerance is a complex trait, there is still a need to analyze its mechanism from a multi-omics perspective. Identifying genes associated with high genetic diversity and local adaptation is highly valuable for pest control. RNAi has practical applications in pest management, yet it's crucial to utilize genetic data to inform our surveillance and intervention strategies. By monitoring *GSC* variation in pest populations, we can detect invasions or outbreak risks. Additionally, targeting associated genetic pathways may help counter pest adaptation and enhance the efficacy of biopesticides, particularly as climates change. This provides new targets for future pest control, using RNAi technology combined with nanomaterials, to further manage and control these pests. To our knowledge, there have been no documented findings on the interactions between cold tolerance and homeobox transcription factors in previous studies. Extensive literature searches using Scopus (https://www.scopus.com/search/form.uri?display=basic#basic) yielded more than 20 000 papers on cold tolerance and over 30 000 papers on homeobox transcription factors. However, none of these papers specifically addressed the correlation between these two aspects in insects. Understanding the variation in both phenotypic and genetic traits among native pest populations is crucial for comprehending colonization dynamics. *C. suppressalis* provides an excellent opportunity to investigate the genetic and evolutionary impacts of local adaptation in widely distributed pests. Our research lays out a strong foundation for guiding practical interventions in pest management. In the context of future pest control, genetic information should guide monitoring and intervention measures. Monitoring genetic variations can help in the early detection of invasions or outbreak risks. Additionally, if pest adaptability is driven by transcribed functional pathways or by targeting on related pathways, technologies like RNAi can be used to develop green nano-pesticides that counteract adaptation or enhance biopesticide efficacy, ultimately achieving effective pest management and control.

Further research is needed to investigate the underlying mechanisms through which reduced expression of *GSC* grants cold tolerance and whether this mechanism applies to other insect species. The identification of this discovery addresses an essential omission in the narrative of pest adaptive evolution and enhances our understanding of the mechanisms that drive rapid adaptation.

## MATERIALS AND METHODS

### Resequencing sample collection

We resequenced 384 individuals, collected in 24 geographic regions (>5 individuals per population) covering its entire distribution in China. These individuals were preserved in 95% ethanol and stored at −80°C until further use. Morphological features and the COI molecular marker were used to identify all *C. suppressalis* individuals, with primers for mtCOI amplification listed in Table S18.

### Extracting and sequencing DNA

To extract genomic DNA from each individual, the DNeasy Blood and Tissue Kit (Qiagen, Hilden, Germany) was employed according to the manufacturer's protocol. Following this, short insertion fragment libraries ranging from 300 to 500 bp were created by BGI-Shenzhen and underwent sequencing on the BGISEQ-T7 platform using standard procedures with 150-bp paired-end (PE) reads. In total, we generated ∼9 Tb resequenced data for further population analysis.

### Read mapping and SNP identification

Initially, SOAPnuke (v.1.5.6) [51] software was used to filter all raw reads with the following parameter: (-n 0.1 -q 0.5 -l 12 -Q 2). Additionally, adapters were trimmed. The resulting high-quality filtered reads were then mapped to the whole *C. suppressalis* genome [21] (mitochondrial and nuclear genome) utilizing Burrows Wheeler Aligner (bwa) (v.0.7.17) [52]. Sorting of these reads was conducted using SAMtools (v.1.7) [53] software, and duplicates were removed via Picard (http://broadinstitute.github.io/picard/). Using the HaplotypeCaller module, variants were detected in each sample, yielding a genomic variant call format (GVCF) file. The variants were detected on a per-chromosome basis, excluding the mitochondrial genome due to its haploid. Subsequently, we integrated the GVCFs of each sample and identified SNPs using the GenotypeGVCFs module with the parameter (-all-sites). Using GATK (v.4.2.3), we collected a total of 121 352 304 raw SNPs by applying filter parameters (QD< 2.0 || MQ< 40.0 || FS >60.0 || MQRankSum < −12.5 || ReadPosRankSum < −8.0). To ensure the quality of our biallelic SNP set, we excluded SNPs with a missing rate greater than or equal to 80% and SNPs with a minor allele of below 0.05 were filtered using VCFtools (v.0.1.12b) [54]. Next, we utilized SnpEff (v.5.0) [55] based on the annotation information of the *C. suppressalis* reference genome to annotate the SNP sets.

### SV calling

Using Delly (v.0.8.3) [56], the SVs were detected by analyzing the mapping results in BAM format obtained from resequencing data. Initially, SV calling was performed individually for each sample and the resulting calls were consolidated into a single VCF file. This combined VCF file was then used to guide a second round of SV calling. SVs that possessed the ‘PASS’ tag, ‘PRECISE’ tag, and genotype of either ‘1/1’ or ‘0/1’ after filtration were chosen for further analysis. Finally, BCFtools (v.1.8) [57] was utilized to merge SVs from all cultivars. The SVs that were identified had to be present in at least three samples.

### Population structure

To avoid the effects of linkage disequilibrium (LD) for ancestry analysis, we further refined the independent loci using plink (v.1.90b4.4) [58] software with parameters ‘window size = 10, step size = 10, and r2 > 0.2’. We utilized fastStructure [59], a variational Bayesian framework, to analyze the population genetic structure and explore values of K ranging from 1 to 20. The optimal K value was determined via chooseK.py in the fastStructure package. To prevent the impact of missing data, high-quality genotypes were imputed with Beagle (v.5.0) [60]. Additionally, we conducted PCAs to avoid genome linkage effects by randomly selecting one SNP from a high-quality SNP dataset in a 5-k region using plink (v.1.90b4.4) [58]. To reduce any influences of natural selection, we also used the neutral dataset (synonymous SNPs) to construct a ML phylogeny with 1000 bootstraps using FastTreeMP (v.2.19) software [61]. These results were also consistent with PCA and ancestry analysis (Fig. S5).

### Genetic diversity and *F*_ST_ statistics

For the evaluation of genetic diversity and *F*_ST_, we randomly sampled the same number of individuals (10 individuals) from each population. Using the high-quality SNP set, we employed VCFtools (v.0.1.12b) [57] to assess nucleotide diversity (π), and population genetic differentiation (*F*_ST_) with 10-kb nonoverlapping windows. We utilized the formula *Nm* = (1− *F*_ST_)/(4*F_ST_) for analyzing the gene flow levels. However, to ensure an accurate estimation of genetic diversity within each population, we utilized the Python library ‘random’ (https://docs.python.org/3/library/random.html) to randomly select 10 individuals. This was done to mitigate any potential influence from variations in population size. We conducted a Mantel test in R 4.0.2 using the vegan package (https://doi.org/cran.r-project.org/web/packages/vegan/index.html) to examine the correlation between genetic distance (*F*_ST_/1−*F*_ST_) [32], geographic distance (km), and environmental distance in populations. The significance of the correlation was determined through 9999 permutations.

### Inference of demographic history

Using SMC++ (v.1.13) [34], demographic history (effective population size and divergence time) was forecasted. To estimate, 10 unphased individuals from each population were chosen and the -m parameter was applied to mask the uncalled regions in the SNP VCF file. To ensure consistent sample sizes from the population, we randomly selected 10 sequencing depths greater than 2× individuals according to the ancestral proportions at K = 5. The VCF file was filtered without considering the minor allele frequency (MAF) parameter.

We further examined the gene flow between peripheral populations and central populations to perform coalescent simulations through the software package fastsimcoal (v.2.7) [62], which estimates parameters using the composite likelihood method. The SNP VCF dataset underwent filtration without the minor allele frequency (MAF) parameter. To avoid the impact of linkage disequilibrium (LD), plink (v.1.90b4.4) was used to extract intergenic SNPs with the parameters ‘window size = 10, step size = 10, and r2 > 0.2’, and the SFS was generated by easySFS.py (https://github.com/isaacovercast/easySFS). According to the findings from SMC++, we developed six models to study population size change. Subsequently, we established gene flow at various time intervals. We applied four models and ran them 50 times to estimate the parameters from 100 000 simulations and 40 conditional maximization (ECM) cycles. The best model was ascertained based on the maximum value of likelihoods and Akaike information criterion. We further performed parametric bootstrap estimations by simulating the *.pv file 100 times, utilizing the parameter estimations of the top model. The mutation rate was preset to 8.4 × 10^−9^ and the yearly generation period was 2.5 generations.

### EEMS projection

We estimated migration surface contours using 800 demes in China. To ensure the convergence of the MCMC chains with default parameters, we ran MCMC analysis for 500 000 iterations and repeated the process with different seeds, including 300 000 burn-in iterations. We used R scripts from the EEMS.PLOT function in the rEEMSplots package (https://github.com/dipetkov/eems) to generate final spatial visualizations depicting migratory surfaces. To assess model robustness, we employed a jackknife sampling approach and conducted EEMS runs after iteratively excluding isolates from a single district.

### Selective sweep analysis

Three approaches, namely z-transformation of *F*_ST_, u-statistics, and the composite likelihood ratio (CLR) method, were employed to identify genomic signatures of selection among distinct populations of *C. suppressalis*. The z transformation of *F*_ST_ with a 10-kb window was calculated by comparing the low latitude population (HN population) to other populations. Using SweeD (v.3.1) [63] with a 10-kb window based on the setting grid size in each chromosome, the CLR was calculated for each population. Candidate regions were identified as areas with the top 5% highest CLR values. Finally, we used RAiSD [64] to identify potential genomic regions that underwent positive selection in the *C. suppressalis* population. The u statistical method incorporated various factors such as genetic diversity, site frequency spectrum (SFS), and linkage disequilibrium when analyzing these regions. The windows were set at a size of 10-kb, and genes falling within the top 5% quantile were identified. Candidate genes were identified after two of the last three methods were used. iHS is used to detect recent positive selection using selscan (v.1.3.0) software [65]. |iHS| >2 in each 100-kbp window, with a slide of 10 kbp, was considered as the candidate region. Using CalcABS software [66], the ABS values were calculated in sliding windows of the genome (a window of 20 kb with a step of 1 kb). To prevent the influence of a low SNP number in certain windows, any window containing fewer than 20 SNPs was excluded. We utilized the formula (HE, EC||SWC, HN) to identify regions of the genome that indicated they were undergoing an ancient selective sweep, where the top 5% of the most extreme values were used as evidence. We utilized these outlier gene regions to identify potential candidate genes through Kyoto Encyclopedia of Genes and Genomes (KEGG) enrichment analysis (https://github.com/GilEshel/OverRep/). FDR correction was performed for the multiple *P* value tests. However, we did not delve into pathways that are relevant to human disease.

### Detection of environment-associated genomic variation

The analysis of genome-environmental associations involved the selection of 19 bioclimatic variables (BIOCLIM) from WorldClim [67] as shown in Table S11. Using the Python library PCA (https://github.com/erdogant/pca), we performed a principal component analysis on these variables and identified PC1, which accounted for ∼83.10% of the bioclimate variation. We observed a strong relationship between latitude and PC1, as depicted in Fig. S13 (R = 0.596, *p* = 0.003). In light of this finding, we selected the latitude and two most important bioclimatic variables that significantly contribute to the *C. suppressalis* population (as determined by Maxent v.3.4.1) [68], and used them for genotype-environment association (GEA) analyses. We employed imputed high-quality genotypes for the linear mixed model (LMM) [69], and applied matrix factorization methods to control for neutral population structure in downstream analyses [70]. LMM was used for GEAs, with both kinship and population structure controlled as structural effects. For population structure estimation, we integrated the PCA outcomes and included the top three components as covariates. Simultaneously, we also excluded two populations (HE and HN) with strong population structure for subsequent analysis. The significance threshold was set at 9.46 × 10^−9^ (0.05/*n*, where *n* is the number of independent SNPs). Additionally, Baypass was also used to identify outlier SNPs using the standard deviation (*SD_XtX* column) of *X^T^X* statistics to identify adaptive variation [71]. The *SD_XtX* value was obtained from the top 99% level. We focused on independent loci to reduce linkage disequilibrium effects using plink (v.1.90b4.4) software with the parameters ‘window size = 10, step size = 20, and r^2^ > 0.2’ [58]. Candidate genes were evaluated by assessing their alignment with adaptive loci or their presence within 2-kb flanking regions on either side. Finally, we identified core adaptive genes and performed KEGG categorization.

### Haplotype analysis of the *GSC* gene

The haplotype analysis matched that of research, as previously described [5]. In summary, the haplotype was constructed using 981 high-quality SNPs from the *GSC* gene. The similarity of variations was calculated using the Python library difflib (https://docs.python.org/3/library/difflib.html). Finally, the order was rearranged based on the similarity score.

### RNA sequencing and differentially expressed gene (DEG) analysis

Using TRIzol reagent (Invitrogen, Carlsbad, USA), total RNA was extracted from five third-instar larvae collected from ten field populations (HLJHH, SDLY, ZJYY, SXHZ, HNYX, SCFS, GZAS, GZGY, GDSZ, and HNSY) according to the manufacturer's protocol. The RNA quality was validated with an NP80 NanoPhotometer (Implen, Munich, Germany) and 1.5% agarose gel electrophoresis. The RNAseq libraries were constructed using the NEBNext^®^ Ultra™ RNA Library Prep Kit (NEB, Ipswich, CA, USA) for Illumina^®^ (Illumina, San Diego, USA) with polyA selection, and the library quality was evaluated using an Agilent Bioanalyzer 2100 system. Finally, the libraries were sequenced on an Illumina NovaSeq platform to generate 150 bp paired-end reads (Novogene, Beijing, China).

Clean reads were obtained and mapped to the *C. suppressalis* genome (http://v2.insect-genome.com/Organism/177) using Hisat2 (v.2.0.5) [72]. The gene expression levels were estimated using the standardized read counts. Three independent biological replicates were performed for each field population.

### Temperature treatment

A total of 150 third-instar larvae were placed in bottles containing fresh rice seedlings. The bottles were then incubated at three different temperatures (18°C, 23°C, and 28°C), and larvae (10 heads per treatment) were collected at four time points (1 h, 2 h, 4 h, and 6 h posttreatment) for RNA extraction. Experiments were independently repeated at least three times.

### RNA extraction, cDNA synthesis, and quantitative real-time PCR analyses

Total RNA was extracted from 10 larvae subjected to different temperature treatments (18°C, 23°C, and 28°C) at various time points (1 h, 2 h, 4 h, and 6 h), including the initial control (0 h), following previously described methods. First-strand cDNA of mRNA was synthesized for quantitative polymerase chain reaction (qPCR) from total RNA using HifairTM 1st Strand cDNA Synthesis SuperMix (Yeasen, Shanghai, China).

Quantitative real-time PCR (RT-qPCR) was conducted using Hieff UNICON^®^ qPCR SYBR Green Master Mix (Yeasen, Shanghai, China) with a CFX96 real-time PCR system to determine mRNA expression levels. The housekeeping genes *EF-1α* and *Actin A1* were utilized as double references for normalizing gene expression levels in *C. suppressalis*. Relative mRNA expression levels were calculated using the 2^–ΔΔCt^ method described by Livak and Schmittgen [73], with each sample analyzed in triplicate. The primers used for RT‒qPCR are listed in Table S18.

### RNAi, supercooling point and net growth rate determination

The specific primers for dsRNA synthesis were designed based on the sequence of the *GSC* gene and the fragment sequence of an enhanced green fluorescent protein (EGFP), which contained the T7 polymerase promoter sequence at both ends (Table S18). EGFP is just a control gene, and its interference will not affect the target gene, but the substance added is the same as the target gene. The PCR products were used as templates for dsRNA synthesis using the T7 RiboMAX Express RNAi System (Promega, Madison, WI, USA). All dsRNAs were resuspended in nuclease-free water and quantified spectrophotometrically. The quantity of dsRNA was checked by agarose gel electrophoresis (1.5%) and an NP80 NanoPhotometer. Then they were stored at −80°C until use. The dsRNA was dissolved in diethyl pyrocarbonate-treated water to a final concentration of 10 μg/μL. The dsRNA (0.4 μL, 4 μg) injection was performed using an Eppendorf InjectMan NI 2 microinjection system (Drummond Company, Inc., Alabama, USA). More than 212 third instar larvae in GDSZ were used for each treatment, subsequently, the interference efficiency of the genes, supercooling point and net growth rate of the larvae were assayed on days 1, 2, and 3 of injection.

The supercooling point at larvae stage was measured as previously described [74]. Treated larvae were placed in a 200 μL pipette tip and fixed with cotton wool. A copper constant thermocouple was attached to the surface of each individual and linked to an automatic temperature recorder (Wuhan TaiWoKang Instrument Equipment Co. Ltd, Wuhan, China). The thermocouple, together with the insect, was placed into a freezer (BC/BD-459HEK, Haier Group Corporation, Shandong). The supercooling point was determined when the temperature recorded by the thermocouple was just before an exothermic event that was caused by the release of the latent heat of crystallization. In total, 14 third instar larvae were tested for each treatment every day.

We determined the net growth rate of larvae by assessing the daily change in their body weight following *GSC* gene silencing treatment at 18°C. In total, 24 third instar larvae were tested for each treatment every day.

## Supplementary Material

nwae221_Supplemental_Files

## Data Availability

The sequence data have been submitted to the National Center for Biotechnology Information (NCBI). The genomic sequence data are available at BioProject PRJNA984920, PRJNA984938, PRJNA985862, PRJNA986824 and PRJNA989397.
