# Supplementary material for: Extreme genetic signatures of local adaptation in a notorious rice pest, Chilo suppressalis
Source: Natl Sci Rev. 2024 Aug 1;12(3):nwae221. doi: 10.1093/nsr/nwae221 (PMC11823119; doi:10.1093/nsr/nwae221)
Supplement: nwae221_Supplemental_Files [file nwae221_supplemental_files.zip › Supplementary Materials.docx]

**Supplementary Materials for**

**Extreme genetic signatures of local adaptation in a notorious rice pest, *Chilo suppressalis***

Yan Peng^1, †^, Kaikai Mao^2,1,†^, Hongran Li^1^, Junfen Ping^1,3,4^, Jingyun Zhu^1^, Xinye Liu^1^, Zhuting Zhang^1^, Minghui Jin^1^, Chao Wu^1^, Nan Wang^1^, Alexander Yesaya^1^,Kenneth Wilson^5,1^, Yutao Xiao^1,*^

^1^Shenzhen Branch, Guangdong Laboratory of Lingnan Modern Agriculture, Key Laboratory of Gene Editing Technologies (Hainan), Ministry of Agriculture and Rural Affairs, Agricultural Genomics Institute at Shenzhen, Chinese Academy of Agricultural Sciences, Shenzhen, China

^2^Guangxi Key Laboratory of Agro-Environment and Agric-Products Safety, College of Agriculture, Guangxi University, Nanning, Guangxi 530004, People's Republic of China

^3^School of Life Sciences, Henan University, Kaifeng 475004, China.

^4^Shenzhen Research Institute of Henan university, Shenzhen 518000, China

^5^Lancaster Environment Centre, Lancaster University, Lancaster, UK

----------------------------------------------------------------------------------------------------------------

***Corresponding author:**

**xiaoyutao@caas.cn (Y.T.X.)**

^†^These authors contributed equally to this work

**The Supplementary Material for the manuscript includes the following:**

Supplementary Figs. S1 to S17

Supplemental Tables notes


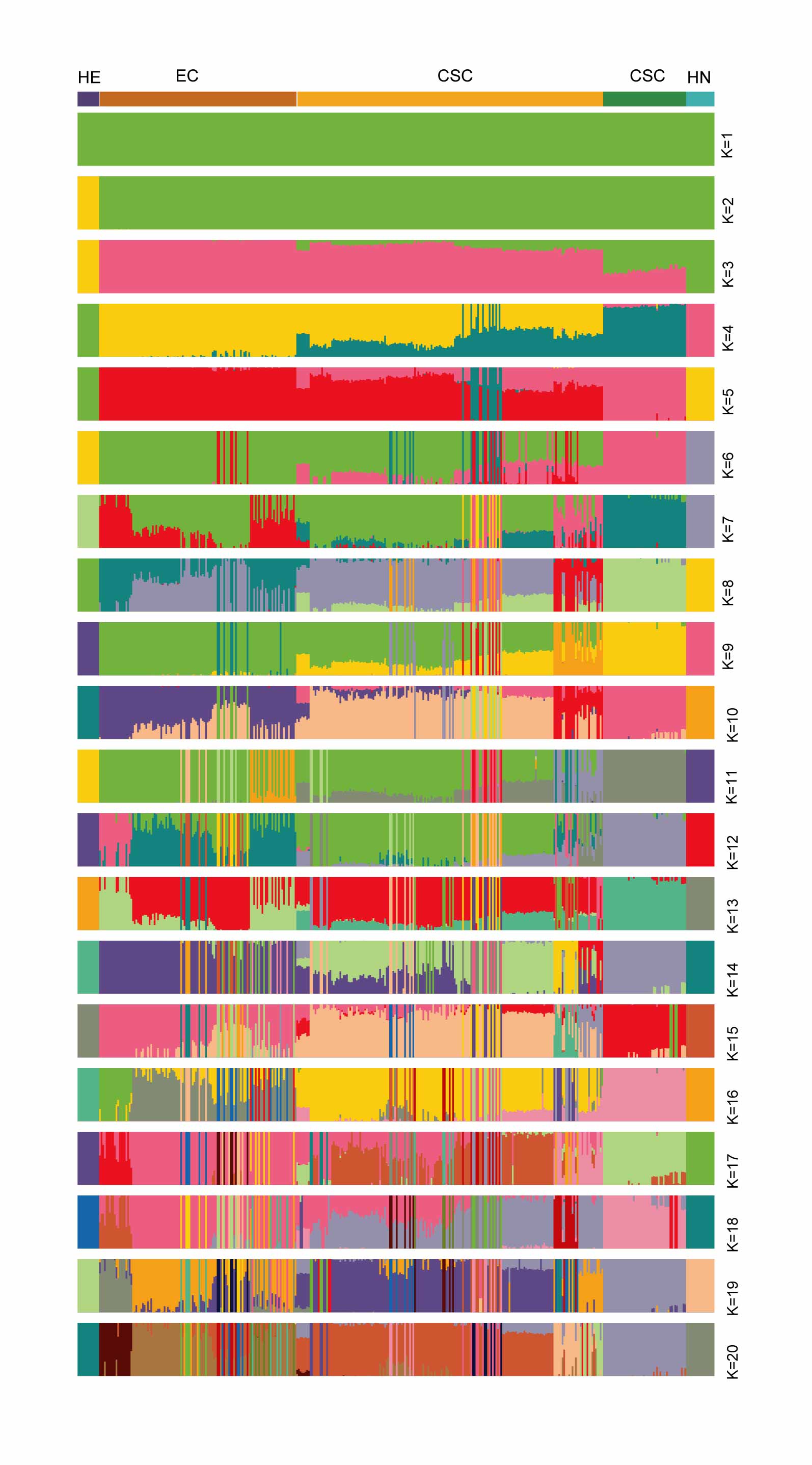


**Supplemental Figure S1.** Population structure of *Chilo su*ppressalis based on SNPs ranging from 2 to 20 using fastStructure software.

**
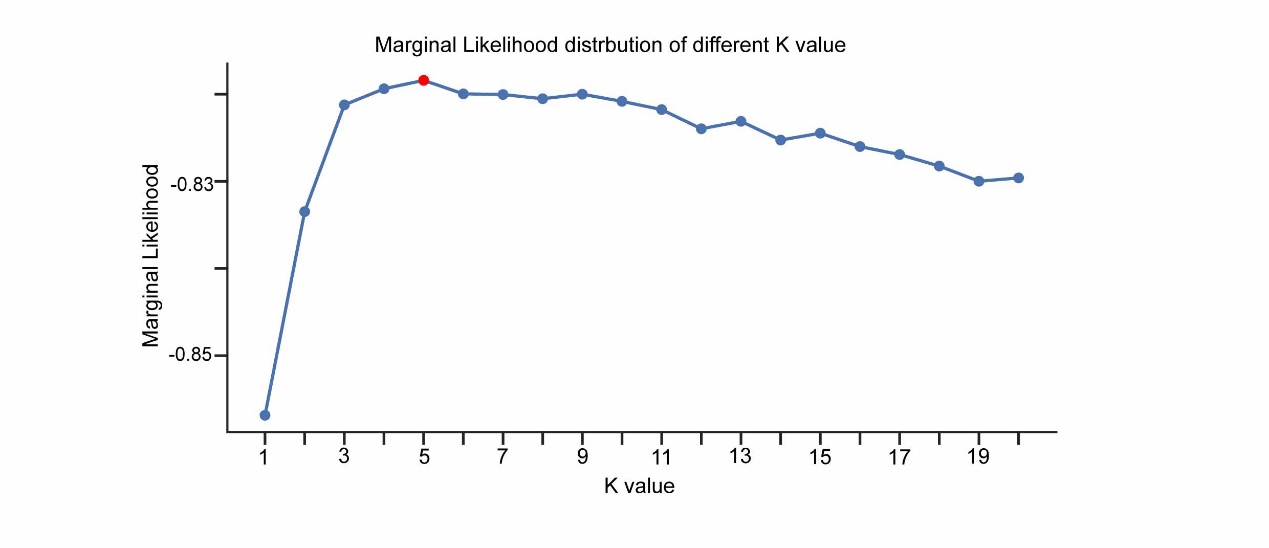
**

**Supplemental Figure S2.** Marginal Likelihood value for different *K* values in fastStructure software. The red color indicates the best *K* value


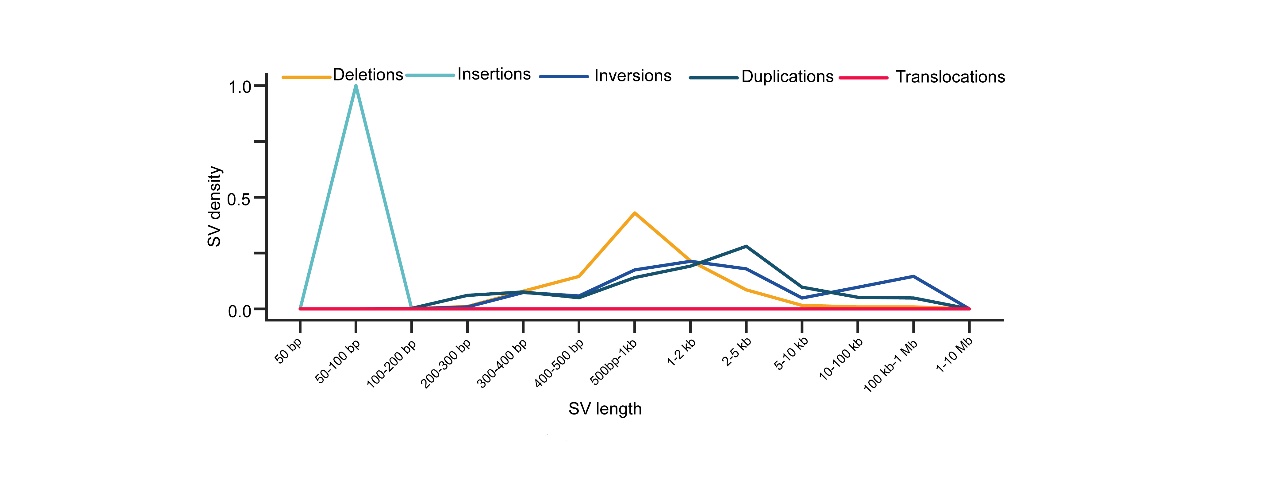


**Supplemental Figure S3.** Structure variation (SV) density of different sizes for each SV type.


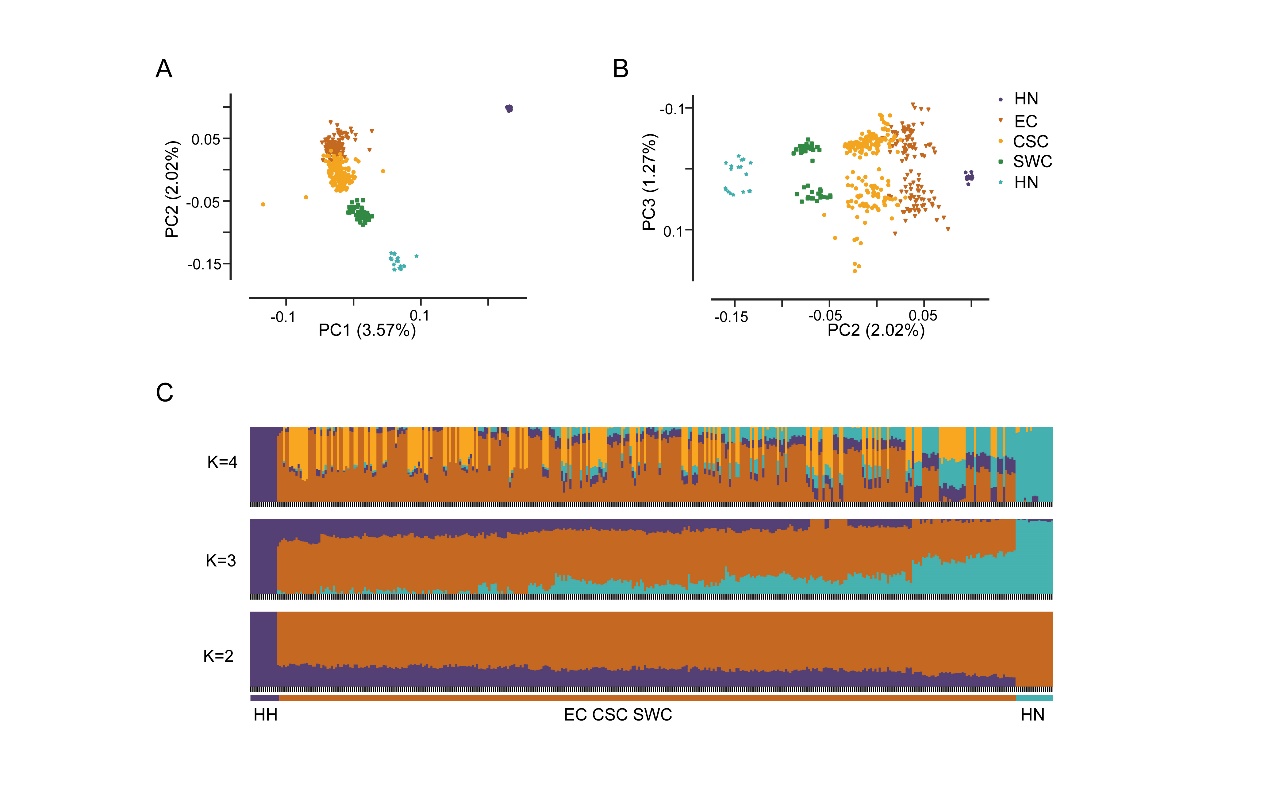


**Supplemental Figure S4. Population structure of *Chilo su*ppressalis based on SVs.** (A-B) PCA plots of *C. su*ppressalis population. (C) Population structure analysis indicating the number of ancestry kinships (K) set ranging from 2 to 4.


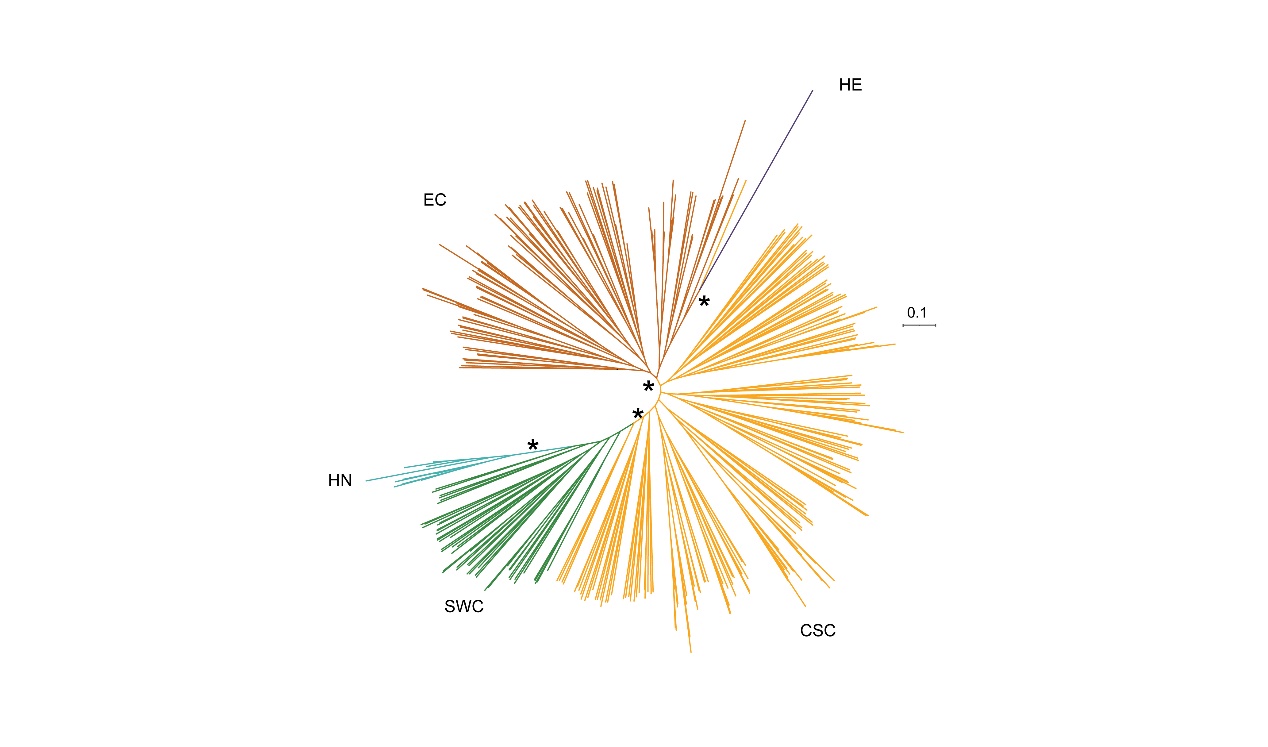


**Supplemental Figure S5. ML tree of *Chilo su*ppressalis.** The tree was inferred from neutral dataset (synonymous SNPs). The values of the bootstrap greater than 90 in main branches were displayed as *.


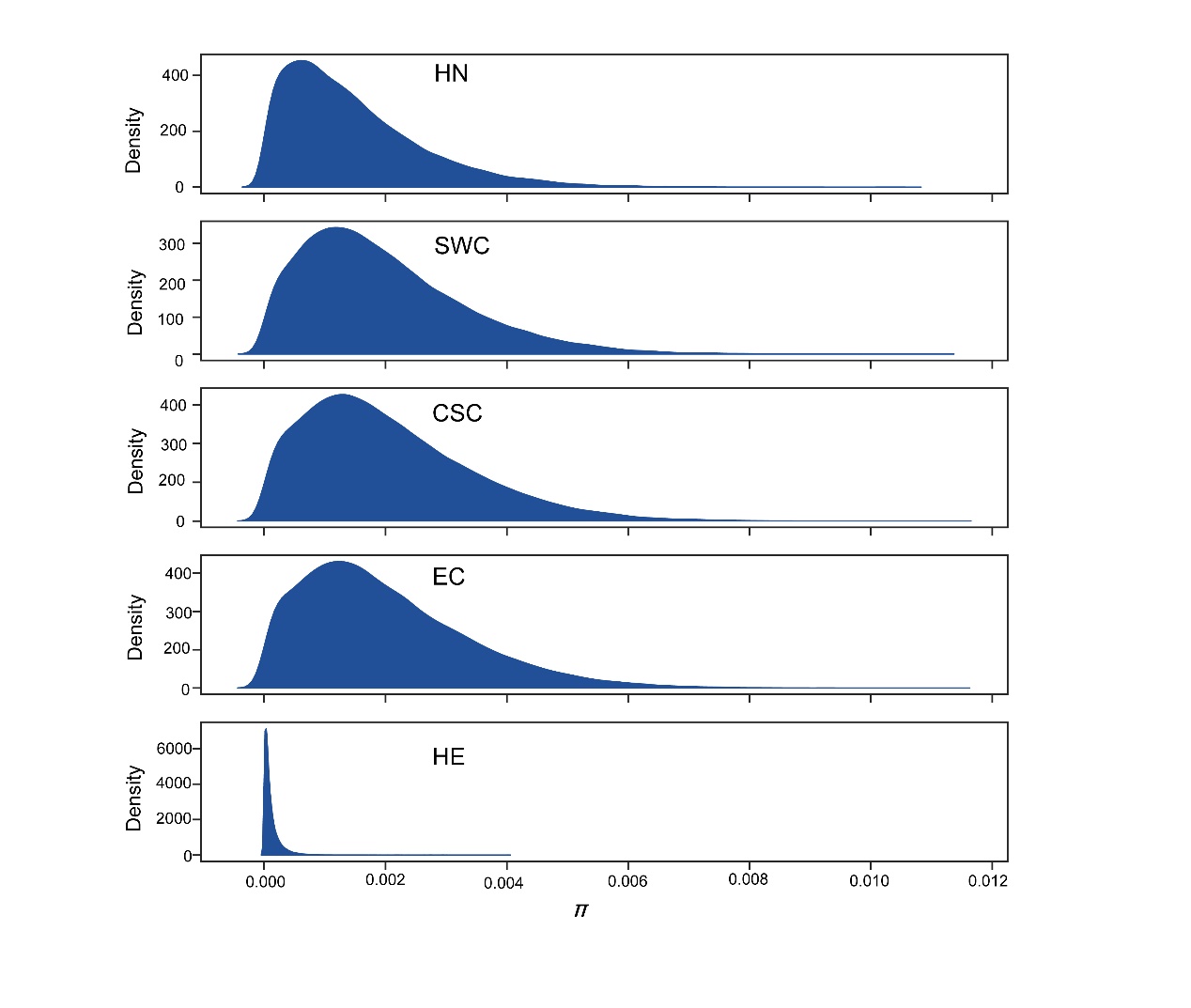


**Supplemental Figure S6.** The density of genetic diversity in *Chilo su*ppressalis population.

**
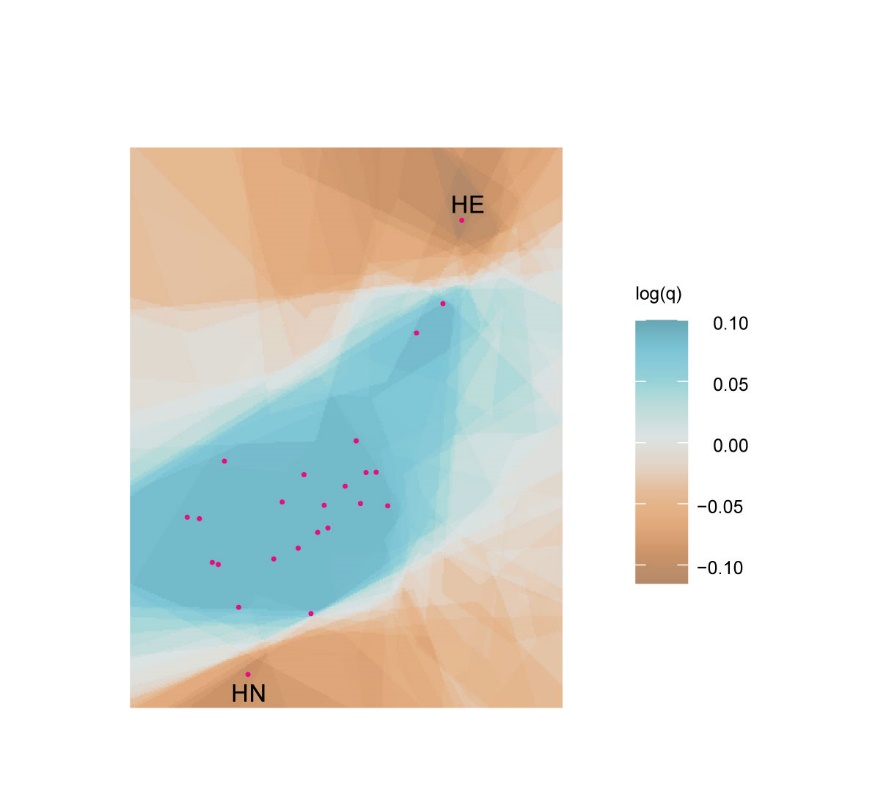
**

**Supplemental Figure S7.** Varying colors show the distribution of genetic diversity in different populations with high (cyan) and low (orange) using EEMS.


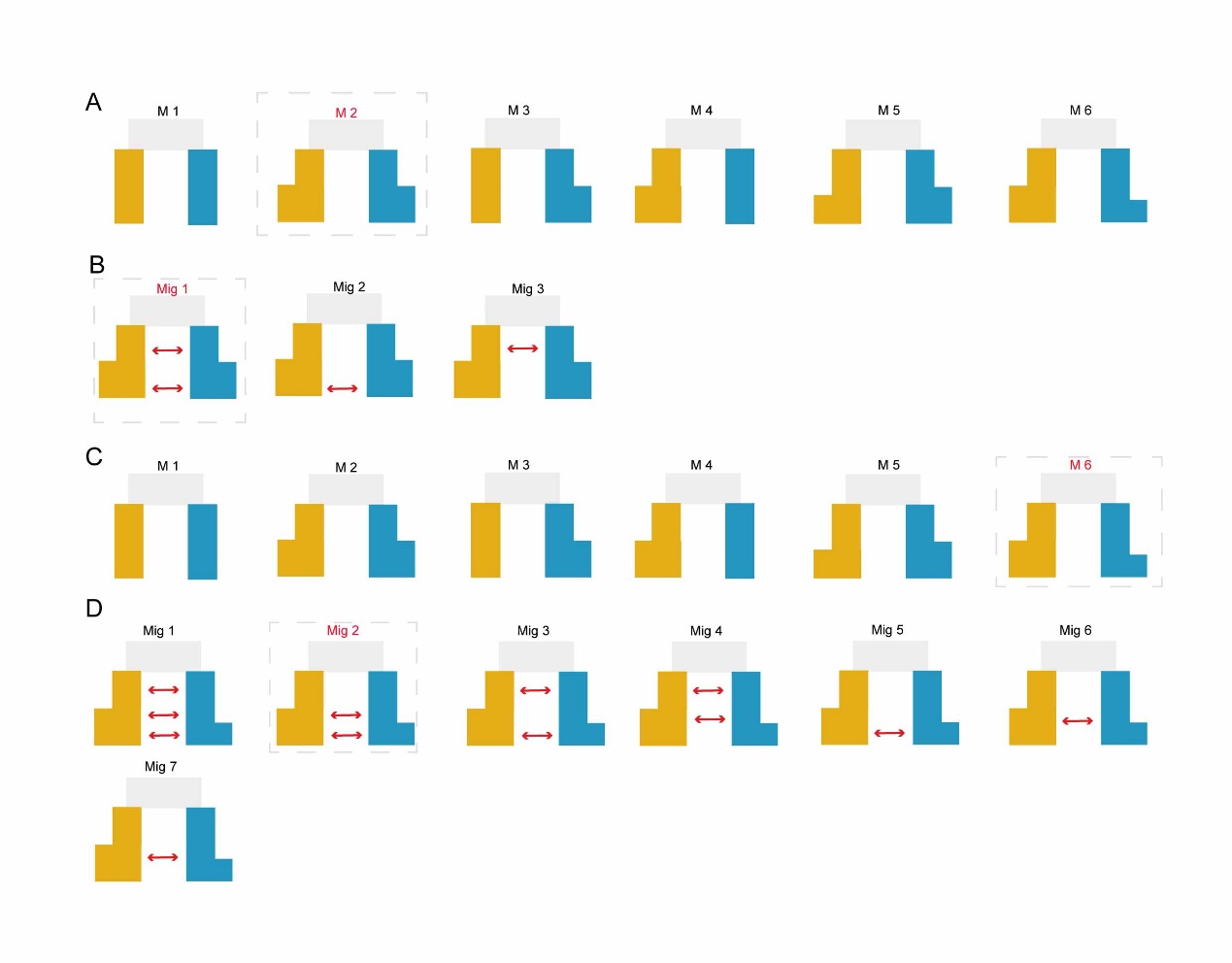


**Supplemental Figure S8. Comparative demographic history of central and peripheral populations.** (A-B) Demographic patterns between central populations (CSC) and peripheral populations (HN). (C-D) Demographic dynamics between central populations (EC) and peripheral populations (HE). The fastsimcoal2 software was utilized to deduce the models for demographic scenarios, with the gene flow being indicated by the arrow. The best model was marked in color.


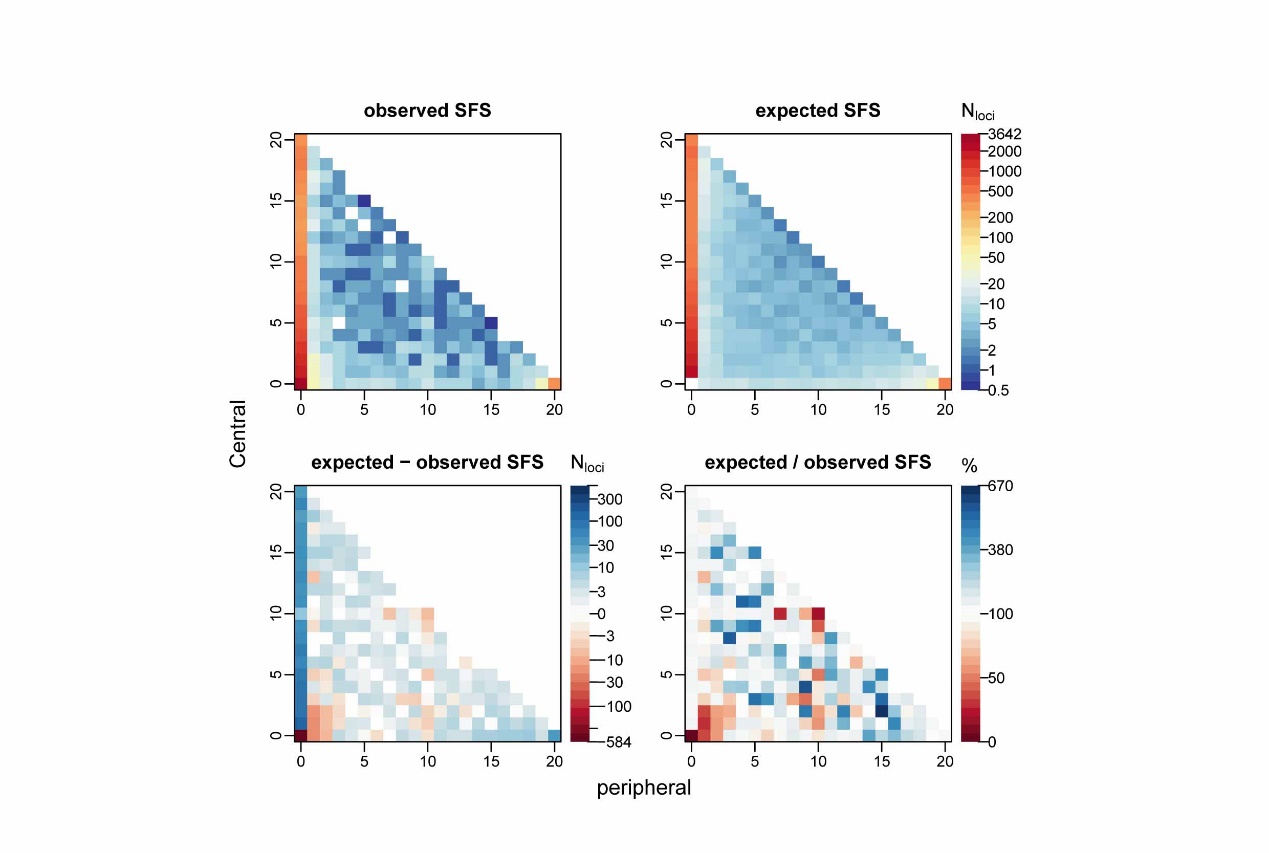


**Supplemental Figure S9.** Observed vs. expected joint 2D-SFS from the best replicate of the scenario for central population and peripheral population (HE).

**
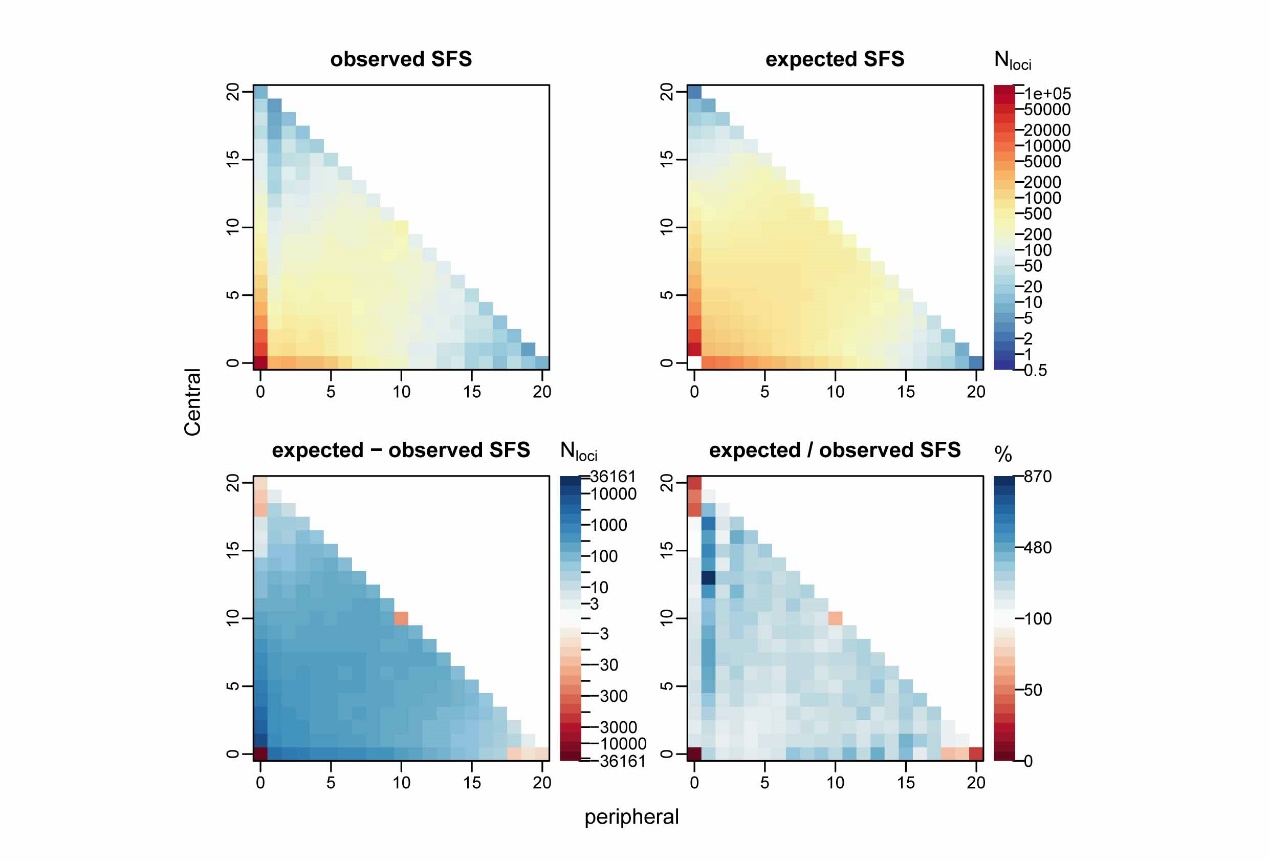
**

**Supplemental Figure S10.** Observed vs. expected joint 2D-SFS from the best replicate of the scenario for central population and peripheral population (HN).

**
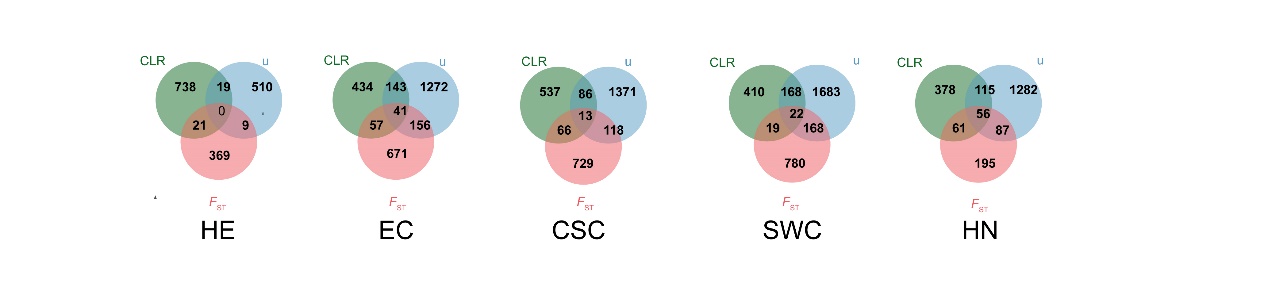
**

**Supplemental Figure S11. V**enn diagrams showing the number of genes identified by selective sweeps using three methods: CLR, u, and Z(*F*_ST_).


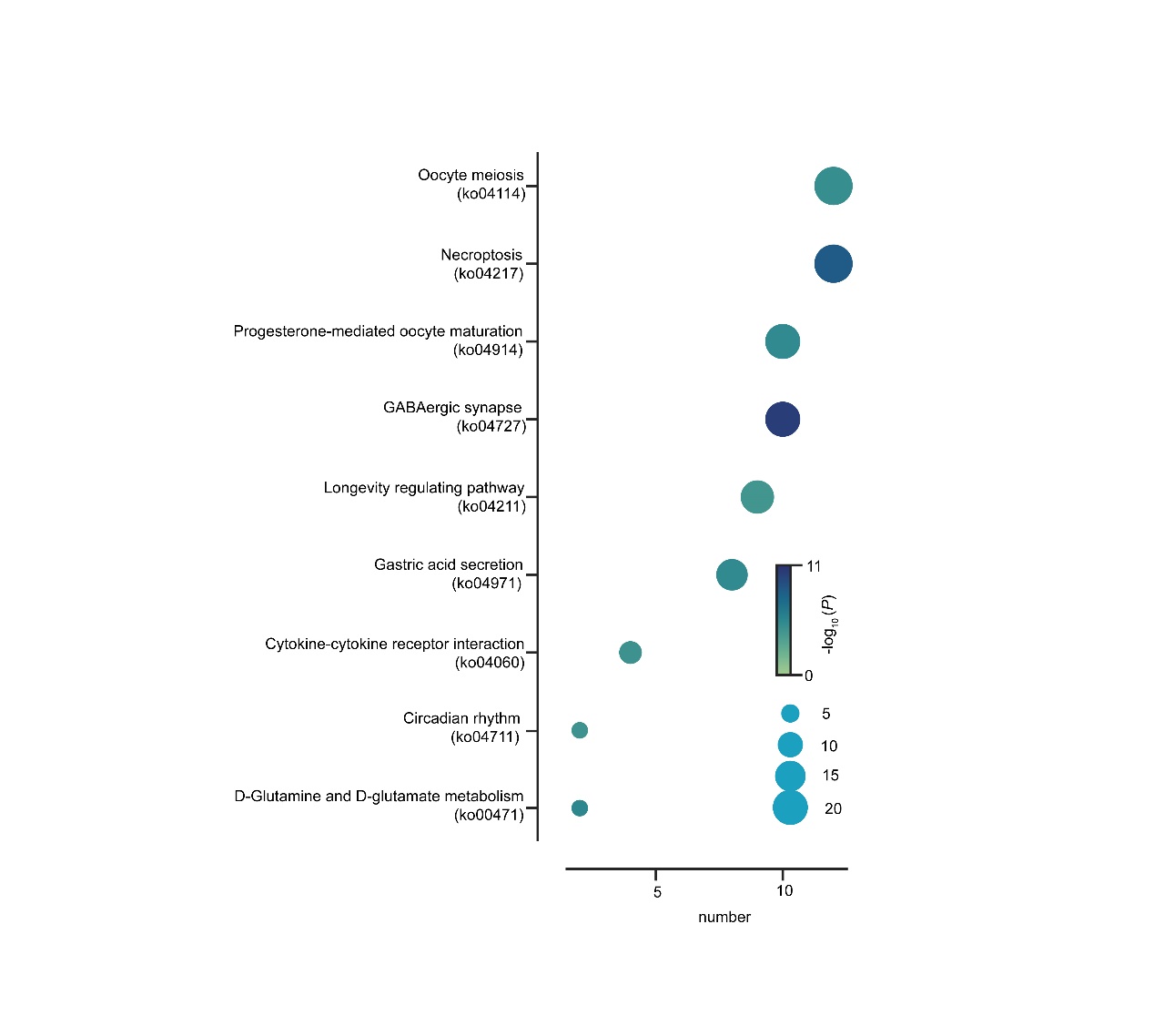


**Supplemental Figure S12. KEGG categorization of selective sweeps identified genes.** The -log(*P*) value is represented by the color of the circle, while the number of selective sweeps signals in the enriched pathway are indicated by the size of the circle.


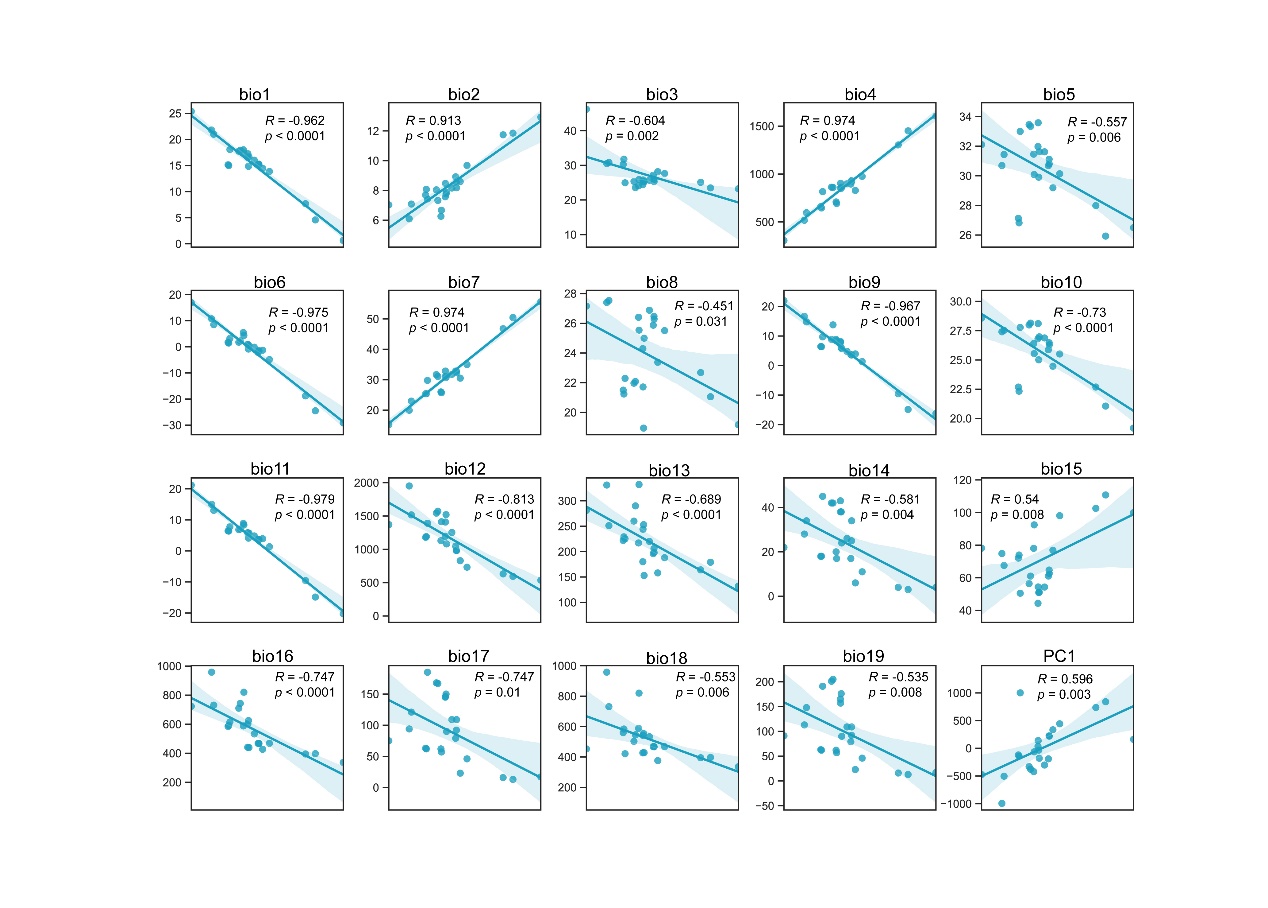


**Supplemental Figure S13. Nineteen environmental factors and principal component (PC1) eigenvectors are associated with latitude.** The relationships between latitude, environmental factors, and PC1 were analyzed using 24 sampling sites.


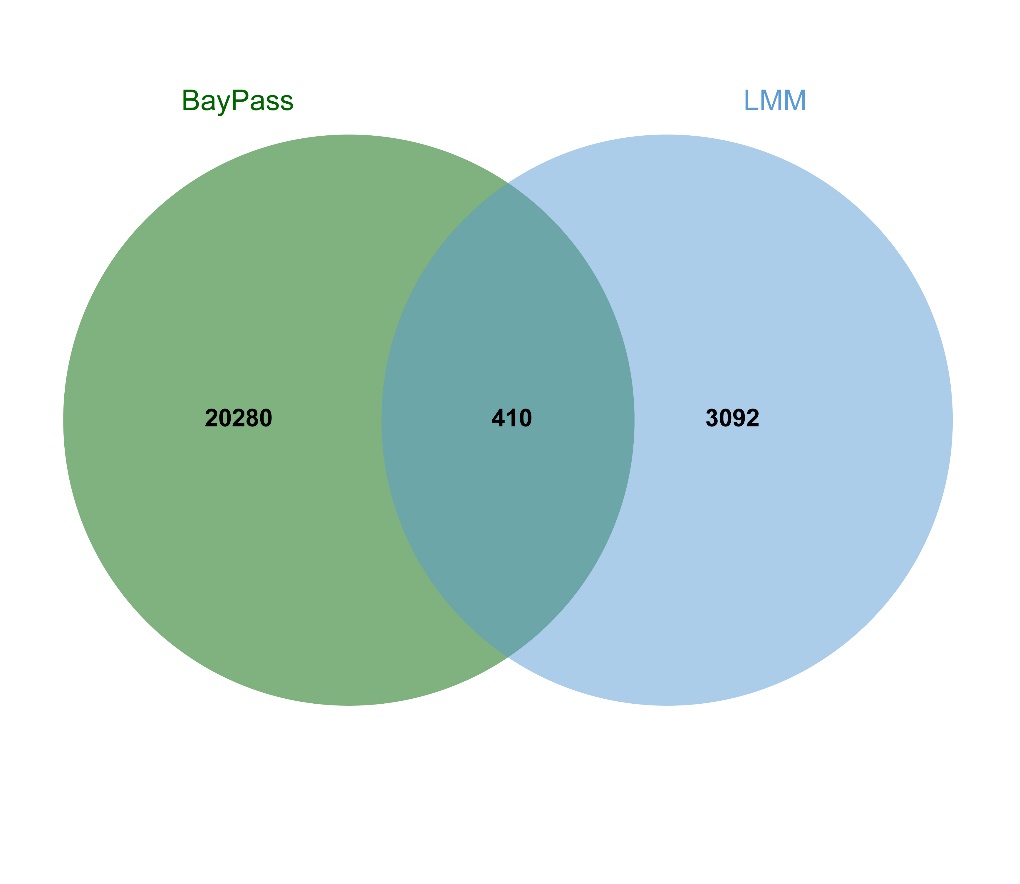


**Supplemental Figure S14.** Venn diagram shows the number of SNPs identified by BayPass and LMM.


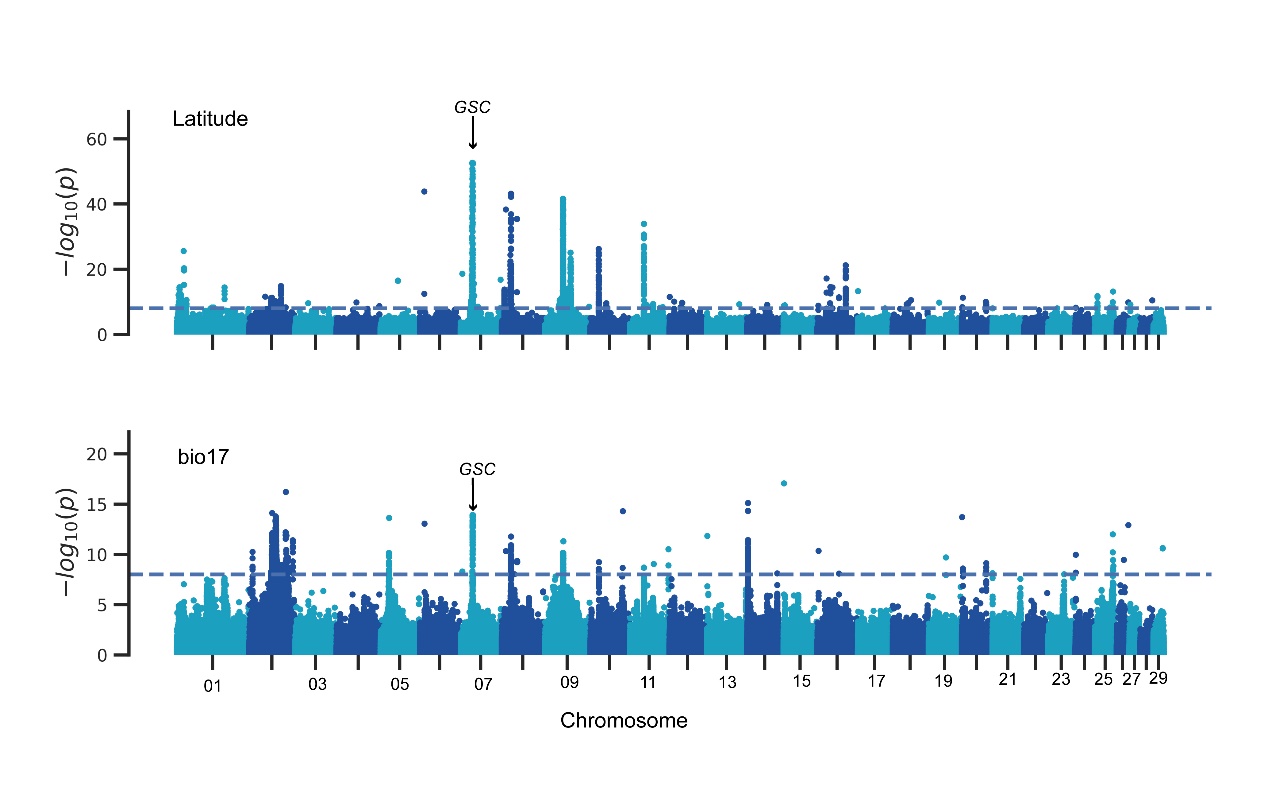


**Supplemental Figure S15.** Manhattan plots show the results associated with latitude and precipitation of the driest quarter (bio17) by LMM.

**
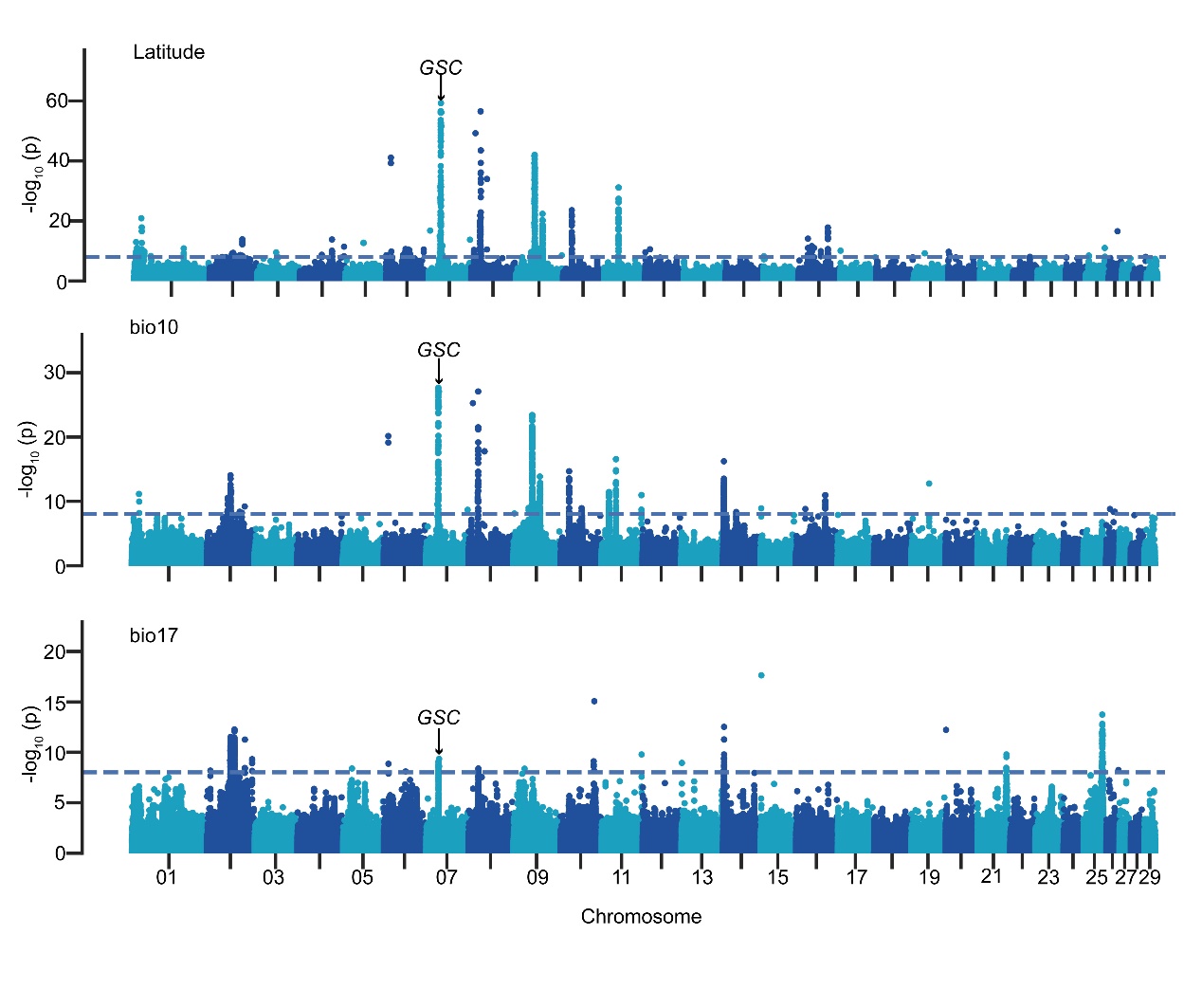
**

**Supplemental Figure S16.** Manhattan plots show the results obtained after excluding the peripheral populations (HE and HN) associated with latitude, mean temperature of the warmest quarter (bio10), and precipitation of the driest quarter (bio17) using LMM.

**
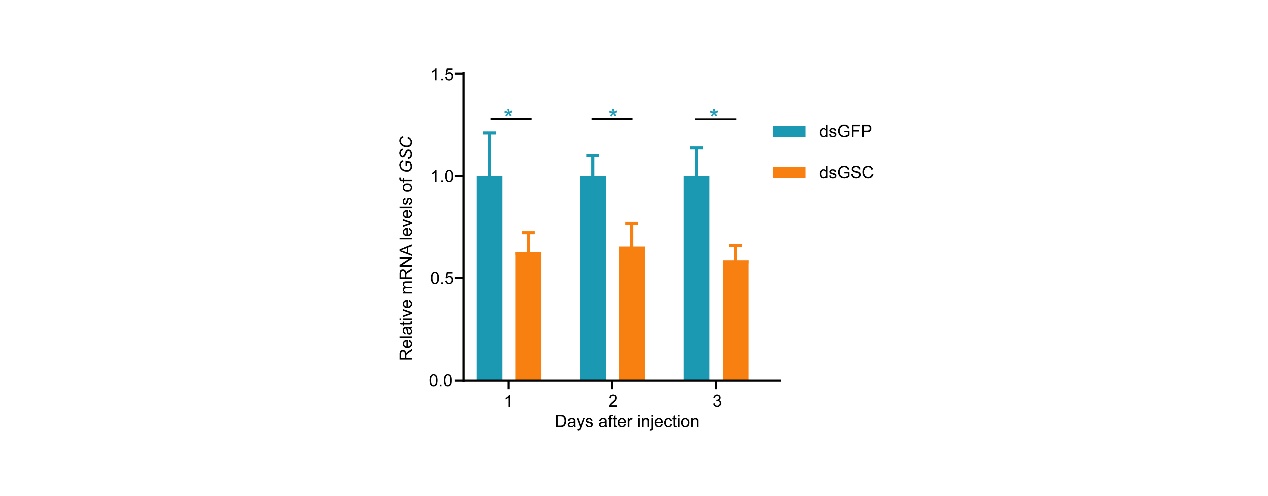
**

**Supplemental Figure S17. The relative expression level of *GSC* after RNAi silencing in *C. suppressalis*.** The *P*-values calculate using Student’s t test, **P*<0.05.

**Supplemental Tables:**

Table S1: The information on the *Chilo suppressalis* collected from different geographical regions.

Table S2: The information on the demographic scenario model between central population and peripheral population.

Table S3: The 95% confidence intervals of the best models between central population and peripheral population.

Table S4: Environmental characteristic and traits summary of the *C. suppressalis* population.

Table S5: The candidate genes of selective sweeps.

Table S6: The selective sweeps results of KEGG enrichment analysis.

Table S7: The candidate genes of iHS.

Table S8: The iHS results of KEGG enrichment analysis.

Table S9: The candidate genes of ABS.

Table S10: The ABS results of KEGG enrichment analysis.

Table S11: The information on bioclimatic variables (bio1–bio19).

Table S12: The contribution of the climatic factor.

Table S13: The results of GEAs do not consider these two peripheral populations (HE and HN) and concentrate only on the primary central populations.

Table S14: The candidate genes of GEAs using LMM.

Table S15: The LMM results of KEGG enrichment analysis.

Table S16: The candidate genes of GEAs using BayPass.

Table S17: The BayPass results of KEGG enrichment analysis.

Table S18: The primers used in this study.
